# Reevaluation of the Phylogenetic Diversity and Global Distribution of the Genus “*Candidatus* Accumulibacter”

**DOI:** 10.1128/msystems.00016-22

**Published:** 2022-04-25

**Authors:** Francesca Petriglieri, Caitlin M. Singleton, Zivile Kondrotaite, Morten K. D. Dueholm, Elizabeth A. McDaniel, Katherine D. McMahon, Per H. Nielsen

**Affiliations:** a Center for Microbial Communities, Department of Chemistry and Bioscience, Aalborg University, Aalborg, Denmark; b Department of Bacteriology, University of Wisconsin—Madison, Madison, Wisconsin, USA; c Microbiology Doctoral Training Program, University of Wisconsin—Madison, Madison, Wisconsin, USA; d Department of Civil and Environmental Engineering, University of Wisconsin—Madison, Madison, Wisconsin, USA; Queen’s University Belfast

**Keywords:** “*Candidatus* Accumulibacter”, diversity, metagenome-assembled genome, phylogeny, *ppk1* gene

## Abstract

“*Candidatus* Accumulibacter” was the first microorganism identified as a polyphosphate-accumulating organism (PAO) important for phosphorus removal from wastewater. Members of this genus are diverse, and the current phylogeny and taxonomic framework appear complicated, with most publicly available genomes classified as “*Candidatus* Accumulibacter phosphatis,” despite notable phylogenetic divergence. The *ppk1* marker gene allows for a finer-scale differentiation into different “types” and “clades”; nevertheless, taxonomic assignments remain inconsistent across studies. Therefore, a comprehensive reevaluation is needed to establish a common understanding of this genus, in terms of both naming and basic conserved physiological traits. Here, we provide this reassessment using a comparison of genome, *ppk1*, and 16S rRNA gene-based approaches from comprehensive data sets. We identified 15 novel species, along with “*Candidatus* Accumulibacter phosphatis,” “*Candidatus* Accumulibacter delftensis,” and “*Candidatus* Accumulibacter aalborgensis.” To compare the species *in situ*, we designed new species-specific fluorescence *in situ* hybridization (FISH) probes and revealed their morphology and arrangement in activated sludge. Based on the MiDAS global survey, “*Ca*. Accumulibacter” species were widespread in wastewater treatment plants (WWTPs) with phosphorus removal, indicating process design as a major driver for their abundance. Genome mining for PAO-related pathways and FISH-Raman microspectroscopy confirmed the potential for PAO metabolism in all “*Ca*. Accumulibacter” species, with detection *in situ* of the typical PAO storage polymers. Genome annotation further revealed differences in the nitrate/nitrite reduction pathways. This provides insights into the niche differentiation of these lineages, potentially explaining their coexistence in the same ecosystem while contributing to overall phosphorus and nitrogen removal.

**IMPORTANCE** “*Candidatus* Accumulibacter” is the most studied PAO, with a primary role in biological nutrient removal. However, the species-level taxonomy of this lineage is convoluted due to the use of different phylogenetic markers or genome sequencing approaches. Here, we redefined the phylogeny of these organisms, proposing a comprehensive approach which could be used to address the classification of other diverse and uncultivated lineages. Using genome-resolved phylogeny, compared to phylogeny based on the 16S rRNA gene and other phylogenetic markers, we obtained a higher-resolution taxonomy and established a common understanding of this genus. Furthermore, genome mining of genes and pathways of interest, validated *in situ* by application of a new set of FISH probes and Raman microspectroscopy, provided additional high-resolution metabolic insights into these organisms.

## INTRODUCTION

Phosphorus (P) removal from wastewater is an essential step in wastewater treatment to prevent environmental damage (e.g., eutrophication) to receiving water bodies. The enhanced biological phosphorus removal (EBPR) process is a cost-effective technology that is increasingly employed for this purpose in wastewater treatment plants (WWTPs) (1, 2). EBPR is mediated by specialized microorganisms known as polyphosphate-accumulating organisms (PAOs), which are able to accumulate P as intracellular polyphosphate (polyP), thereby allowing the removal of excess P from the water by disposing of surplus sludge (1). One of the first PAOs identified, which is still considered the model PAO organism, was “*Candidatus* Accumulibacter” from the Rhodocyclaceae family in the Proteobacteria (3, 4).

“*Ca.* Accumulibacter” species have not been isolated in pure culture, despite enrichment efforts (5, 6). Cultivation-independent approaches have been essential and have been widely applied to investigate these microorganisms (4, 7–12), and “*Ca*. Accumulibacter” populations have shown to be abundant both in lab-scale EBPR reactors (4, 13) and in full-scale EBPR plants (8, 14, 15). However, the 16S rRNA marker gene, a common target for culture-independent techniques, is highly conserved within the genus, which prohibits the differentiation of functionally important subgenus taxa (i.e., species or strains) (16). To overcome this problem, the phylogeny of “*Ca.* Accumulibacter” has been resolved by sequencing of the polyphosphate kinase gene (*ppk1*) (8, 17, 18), which encodes an enzyme involved in polyP accumulation (16). Using the *ppk1* gene, the genus has been grouped into two major divisions, type I and type II, each with multiple subdivisions referred to as clades (clades IA to IH and IIA to II_i) (7, 16, 17). It has generally been assumed that this dichotomy could mirror the phenotypic variants observed under different environmental conditions. The most contradictory of these differences was the ability of “*Ca*. Accumulibacter” clades to couple P uptake with nitrate reduction, with a general agreement that only type I can uptake P using nitrate as an electron acceptor, whereas type II cannot (5, 13, 19). However, respiratory nitrate reduction was later observed in lab-scale reactors enriched with “*Ca*. Accumulibacter” clade IIC (20). Other studies have also suggested that, despite both types being able to adopt a glycogen-accumulating organism (GAO) metabolism under P-limiting conditions, the metabolic flexibility of “*Ca*. Accumulibacter” type II would give it a competitive advantage under such conditions (21–23).

These discrepancies have motivated efforts to use comparative genomics to define key traits at finer scales of resolution. This was first achieved by García Martín et al. (11) with the subsequent completion of the genome for “*Ca*. Accumulibacter” clade IIA strain UW-1. Recently, the application of high-throughput sequencing techniques has allowed the recovery of thousands of high-quality metagenome-assembled genomes (MAGs) from WWTP ecosystems (24, 25), providing an excellent opportunity to investigate the diversity and ecophysiology of microorganisms important in these systems, including those of “*Ca*. Accumulibacter.” Genome-based approaches are also an invaluable instrument to resolve the phylogeny of this microbial group. Even though *ppk1*-based phylogeny and genome-based taxonomy generally coincide, a recent study from McDaniel et al. (12) observed some discrepancies, with a few MAGs classified as “*Ca*. Accumulibacter” branching outside the established taxonomy. Moreover, most of the publicly available “*Ca.* Accumulibacter”-associated genomes are currently classified as “*Candidatus* Accumulibacter phosphatis,” despite notable phylogenetic divergence, increasing confusion in the taxonomic assignments and highlighting the need for a substantial reevaluation of the phylogeny of this genus.

This confusion is also evident when using fluorescence *in situ* hybridization (FISH) to study the morphological diversity within this genus. Three FISH probes (PAOmix probes) were designed to target their 16S rRNA (4). However, the PAOmix has been shown to be inadequate to specifically distinguish “*Ca.* Accumulibacter” from other phylogenetically related taxa, and it targets species belonging to the genus Propionivibrio, a well-known GAO (26). Another FISH probe set was designed more recently to distinguish type I from type II (19) and displays a morphologically heterogeneous community. Therefore, a reevaluation of existing FISH probes targeting “*Ca.* Accumulibacter” is needed for confident application in future studies. This could benefit from the use of comprehensive and ecosystem-specific full-length 16S rRNA gene reference databases, such as MiDAS3 (27, 28) and MiDAS4 (29), which facilitate the analysis of the microbial diversity in WWTPs with species-level resolution.

Here, we used a collection of new and publicly available MAGs to obtain a comprehensive comparison of genome-, *ppk1*-, and 16S rRNA gene-based phylogenies to redefine the taxonomy of the “*Ca*. Accumulibacter” genus. Microbial community data from the MiDAS global project was used to profile the abundance and distribution of “*Ca.* Accumulibacter” species in full-scale WWTPs worldwide through 16S rRNA gene amplicon sequencing. The full-length 16S rRNA gene sequences were used to reevaluate existing FISH probes and to design a set of new species-level FISH probes to determine their morphology and abundance. The FISH probes were applied in combination with Raman microspectroscopy to detect the storage polymers typical of the PAO metabolism, and these main metabolic traits were subsequently confirmed by annotation of key genes for polyP, glycogen, and polyhydroxyalkanoates (PHA) accumulation, as well as for nitrogen metabolism. Using this approach, we identified 18 novel “*Ca*. Accumulibacter” species, for which we provide here “*Candidatus*” names, and substantially resolved the complex phylogeny of this lineage.

## RESULTS AND DISCUSSION

### Reevaluation of the phylogeny of the genus “*Ca.* Accumulibacter” and other related *Rhodocyclaceae* family members.

**FIG 1:**
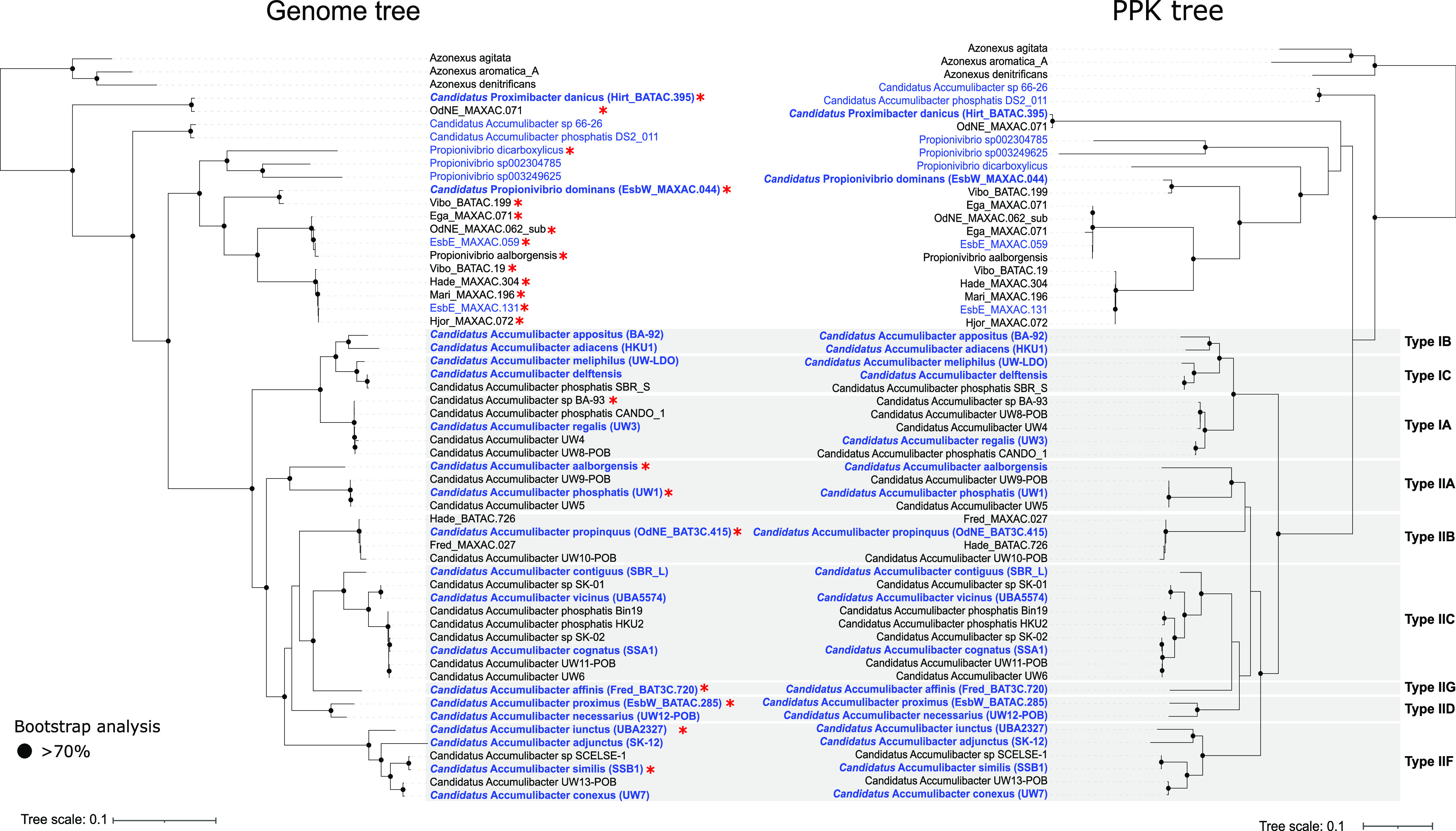
Comparison between genome- and *ppk1*-based phylogenies. The maximum-likelihood genome tree was created from the concatenated alignment of 120 single-copy marker gene proteins trimmed to 5,000 amino acids using GTDB-Tk and 100 bootstraps. Three *Azonexus* (formerly *Dechloromonas*) isolates (GenBank assembly accession numbers IMG taxon_id 637000088, GCA_000519045.1, and GCA_001551835.1) were used as an outgroup. The maximum-likelihood *ppk1* gene tree was created from the alignment of the *ppk1* genes extracted from the genomes, using 100 bootstraps. For NCBI GenBank genome accession numbers and leaf names, see Data File S1 (available at https://doi.org/10.6084/m9.figshare.17306771.v1). Gray boxes indicate the *ppk1*-based types nomenclature. Species representatives are indicated in blue. Of these, the ones with established or proposed “*Candidatus*” names are indicated in bold. Red asterisks indicate MAGs with full-length 16S rRNA gene sequences.

Seventeen MAGs with either Genome Taxonomy Database (GTDB) taxonomy or MiDAS3 16S rRNA gene classification as “*Ca*. Accumulibacter” or Propionivibrio were identified in a set of 1,083 high-quality (HQ) MAGs recovered from Danish WWTPs (25). These genome sequences were added to a collection of publicly available MAGs (12, 24, 30–33) meeting the completeness and contamination quality thresholds for HQ MAGs (>90% completeness and <5% contamination) to obtain a comprehensive overview of the phylogenetic relationship between known “*Ca.* Accumulibacter” taxa and related Rhodocyclaceae family members and to resolve the classification of these genera. Phylogenomic analysis based on conserved marker genes (Fig. 1) revealed distinction into several different genera, as follows: “*Ca.* Accumulibacter”, Propionivibrio, Azonexus (formerly Dechloromonas), and a previously undescribed genus. A total of 36 MAGs retrieved from complex communities, often representing a mixture of several strains (34), clustered within the genus “*Ca*. Accumulibacter,” and, based on the proposed genome-wide average nucleotide identity (ANI) cutoff of 95% for species (35, 36), we identified representatives for 18 species (Fig. 1). Only two of these matched the previously described species “*Ca.* Accumulibacter aalborgensis” and “*Ca.* Accumulibacter delftensis.” While GTDB-based taxonomy recognized many of the MAGs as different species, it still identified the majority as “*Candidatus* Accumulibacter phosphatis,” with an appended letter to distinguish them because of the lack of proposed names (Fig. 1; see also Fig. S1 in the supplemental material).

10.1128/msystems.00016-22.1 FIG S1 Pairwise genome-wide average nucleotide identity (ANI) comparisons of “*Candidatus* Accumulibacter” references. Download FIG S1, PDF file, 0.4 MB. Copyright © 2022 Petriglieri et al. 2022 Petriglieri et al. https://creativecommons.org/licenses/by/4.0/ This content is distributed under the terms of the Creative Commons Attribution 4.0 International license.

In the first instance, we proposed “*Candidatus*” names for the species identified based on genome-phylogeny which were also linked to full-length 16S rRNA gene sequences. Of the 18 species-representative MAGs, only six possessed full-length 16S rRNA gene sequences. Among these, we propose the MAG “*Candidatus* Accumulibacter phosphatis” UW1 (11) as the genus representative, as the highest-quality and first MAG retrieved for this genus, and we assigned it the species name “*Candidatus* Accumulibacter phosphatis.” For the remaining five species, we propose the names “*Candidatus* Accumulibacter propinquus,” “*Candidatus* Accumulibacter affinis,” “*Candidatus* Accumulibacter proximus,” “*Candidatus* Accumulibacter iunctus,” and “*Candidatus* Accumulibacter similis” (Fig. 1 and Table 1). However, as the genome- and *ppk1*-based phylogenies were largely concordant (Fig. 1 and Fig. S1), we can confidently propose names for the remaining species representatives despite the lack of the 16S rRNA gene sequences in the MAGs (Fig. 1 and Table 1).

**TABLE 1:** Different phylogenetic taxonomies of species in “*Ca*. Accumulibacter” and related genera^*c*^

MAG identifier^*a*^	*ppk*-based classification	MiDAS4 classification and ASV details (100% identity)	Species name^*b*^
*Candidatus* Accumulibacter phosphatis UW3	IA	—	“*Ca.* Accumulibacter regalis” UW3
*Candidatus* Accumulibacter sp. BA-93	IA	—	“*Ca.* Accumulibacter regalis” BA-93
*Candidatus* Accumulibacter phosphatis CANDO_1	IA	—	“*Ca.* Accumulibacter regalis” CANDO_1
*Candidatus* Accumulibacter UW4	IA	—	“*Ca.* Accumulibacter regalis” UW4
*Candidatus* Accumulibacter UW8-POB	IA	—	“*Ca.* Accumulibacter regalis” UW8-POB
*Candidatus* Accumulibacter sp. BA-92	IB	—	“*Ca.* Accumulibacter appositus” BA-92
*Candidatus* Accumulibacter phosphatis HKU1	IB	—	“*Ca.* Accumulibacter adiacens” HKU1
*Candidatus* Accumulibacter UW-LDO	IC	—	“*Ca.* Accumulibacter meliphilus” UW-LDO
*Candidatus* Accumulibacter delftensis	IC	“*Ca.* Accumulibacter aalborgensis” (ASV402, ASV1099, ASV1223, ASV2139)	“*Ca.* Accumulibacter delftensis”
*Candidatus* Accumulibacter phosphatis SBR_S	IC	—	“*Ca.* Accumulibacter delftensis” SBR_S
*Candidatus* Accumulibacter aalborgensis	IIA	“*Ca.* Accumulibacter aalborgensis” (ASV771, ASV2139)	“*Ca.* Accumulibacter aalborgensis”
*Candidatus* Accumulibacter phosphatis UW1	IIA	“*Ca.* Accumulibacter phosphatis” (ASV402, ASV1099, ASV1223, ASV2139)	“*Ca.* Accumulibacter phosphatis” UW1
*Candidatus* Accumulibacter UW9-POB	IIA	—	“*Ca.* Accumulibacter phosphatis” UW9-POB
*Candidatus* Accumulibacter UW5	IIA	—	“*Ca.* Accumulibacter phosphatis” UW5
OdNE_BAT3C.415	IIB	“*Ca.* Accumulibacter phosphatis” (ASV548, ASV865)	“*Ca.* Accumulibacter propinquus”
Hade_BATAC.726	IIB	“*Ca.* Accumulibacter phosphatis” (ASV548, ASV865)	“*Ca.* Accumulibacter propinquus”
Fred_MAXAC.027	IIB	“*Ca.* Accumulibacter phosphatis” (ASV548, ASV865)	“*Ca.* Accumulibacter propinquus”
*Candidatus* Accumulibacter UW10-POB	IIB	—	“*Ca.* Accumulibacter propinquus” UW10-POB
*Candidatus* Accumulibacter phosphatis SBR_L	IIC	—	“*Ca.* Accumulibacter contiguus” SBR_L
*Candidatus* Accumulibacter sp. SK-01	IIC	—	“*Ca.* Accumulibacter vicinus” SK-01
Accumulibacter phosphatis_E UBA5574	IIC	—	“*Ca.* Accumulibacter vicinus” UBA5574
*Candidatus* Accumulibacter phosphatis Bin19	IIC	—	“*Ca.* Accumulibacter cognatus” Bin19
*Candidatus* Accumulibacter phosphatis HKU2	IIC	—	“*Ca.* Accumulibacter cognatus” HKU2
*Candidatus* Accumulibacter sp. SK-02	IIC	—	“*Ca.* Accumulibacter cognatus” SK-02
*Candidatus* Accumulibacter phosphatis SSA1	IIC	—	“Ca. Accumulibacter cognatus” SSA1
*Candidatus* Accumulibacter UW11-POB	IIC	—	“*Ca.* Accumulibacter cognatus” UW11-POB
*Candidatus* Accumulibacter UW6	IIC	—	“*Ca.* Accumulibacter cognatus” UW6
EsbW_BATAC.285	IID	“*Ca.* Accumulibacter phosphatis” (ASV548)	“*Ca.* Accumulibacter proximus”
*Candidatus* Accumulibacter UW12-POB	IID	—	“*Ca.* Accumulibacter necessarius” UW12-POB
*Candidatus*_Accumulibacter_B UBA2327	IIF	“*Ca.* Accumulibacter” midas_s_12920 (ASV908)	“*Ca.* Accumulibacter iunctus” UBA2327
*Candidatus* Accumulibacter sp. SK-12	IIF	—	“*Ca.* Accumulibacter adjunctus” SK-12
*Candidatus* Accumulibacter sp. SCELSE-1	IIF	—	“*Ca.* Accumulibacter similis” SCELSE-1
*Candidatus* Accumulibacter phoshatis SSB1	IIF	“*Ca.* Accumulibacter” midas_s_12920 (ASV908)	“*Ca.* Accumulibacter similis” SSB1
*Candidatus* Accumulibacter UW13-POB	IIF	—	“*Ca.* Accumulibacter conexus” UW13-POB
*Candidatus* Accumulibacter UW7	IIF	—	“*Ca.* Accumulibacter conexus” UW7
Fred_BAT3C.720	IIG	“*Ca.* Accumulibacter phosphatis” (ASV548)	“*Ca.* Accumulibacter affinis”
Hirt_BATAC.395	—	“*Ca.* Accumulibacter” midas_s_168 (ASV154, ASV471)	“*Ca.* Proximibacter danicus”
EsbW_MAXAC.044	—	“*Ca.* Accumulibacter” midas_s_315 (ASV124, ASV600)	“*Ca.* Propionivibrio dominans”

a As shown in the genome tree.

b New names proposed in this study.

c —, not applicable.

The genome-resolved phylogeny broadly mirrored the “type” division based on *ppk1* phylogeny commonly used for the “*Ca.* Accumulibacter” genus (Fig. 1). According to the ANI analysis (Fig. S1), “*Ca.* Accumulibacter” MAGs within individual *ppk1*-defined clades fell within the >95% ANI cutoff, while type I and II genomes were similar by approximately 80 to 85% ANI, as recently observed by McDaniel et al. (12). Based on these results and the proposed genus ANI boundary of 75 to 77% (35, 36), there is no evidence supporting the division of type I and type II genomes into separate genera. However, the dichotomy between the two types seem to indicate a phylogenetically relevant clustering into clades and could still be useful for future studies to define different clusters at the interspecies level.

**FIG 2:**
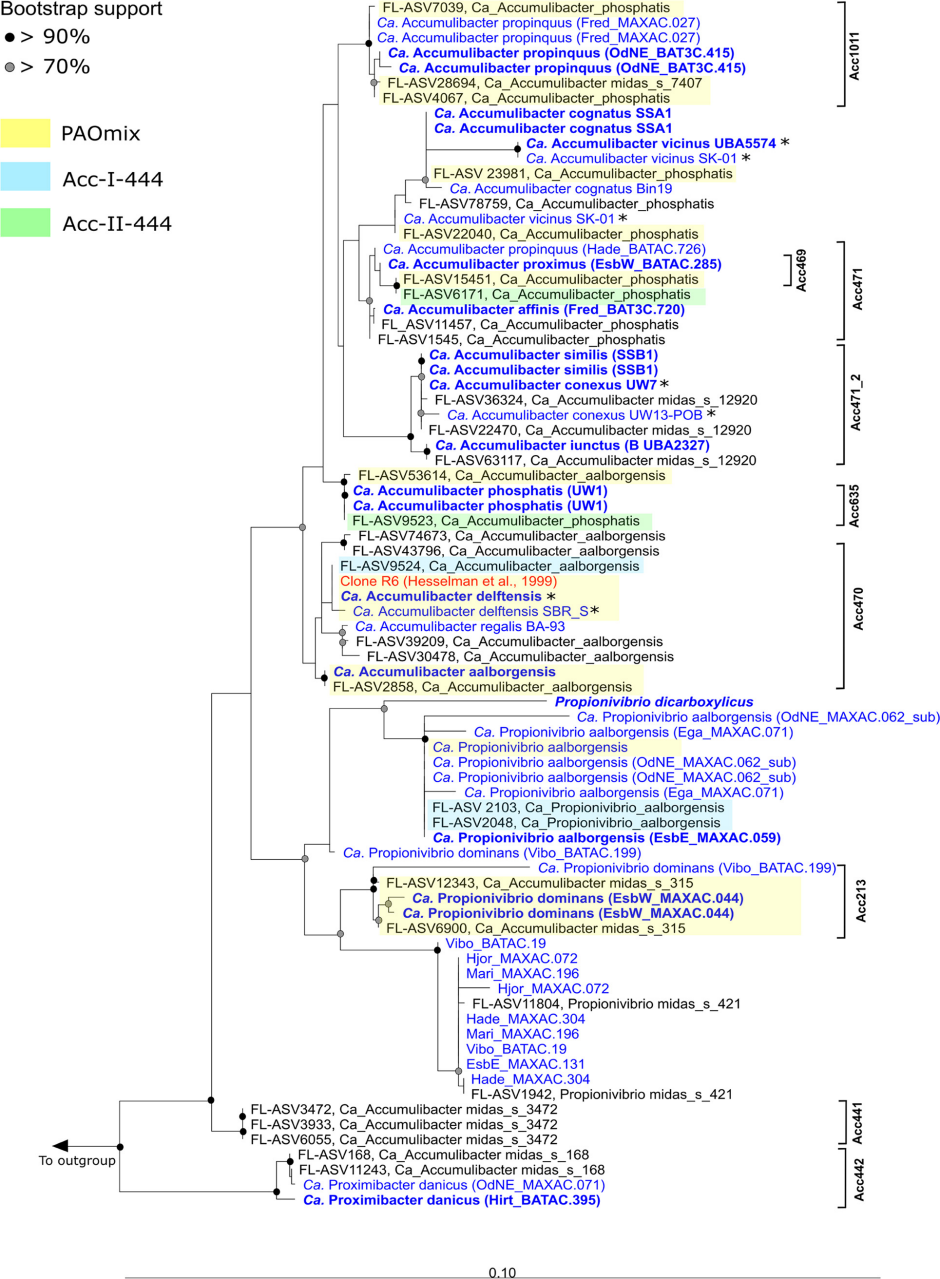
Maximum-likelihood (PhyML)16S rRNA gene phylogenetic tree of “*Ca.* Accumulibacter” and related species. 16S rRNA gene sequences retrieved from the MAGs are indicated in blue, the original 16S rRNA gene sequence (GenBank accession number AJ224937) retrieved from Hesselman et al. (3) is indicated in red. The species renamed in this study are indicated in bold blue. 16S rRNA gene partial sequences are indicated with a black asterisk. The alignment used for the tree applied a 20% conservational filter to remove hypervariable positions, giving 1,250 aligned positions. Coverage of the fluorescence *in situ* hybridization (FISH) probes designed in this study is indicated with black brackets and is based on the MiDAS4 database (29). Probe coverage of widely applied probes for the “*Ca.* Accumulibacter” clades is shown with yellow (PAO651), orange (Acc-I-44), and red boxes (Acc-II-444). Bootstrap values from 1,000 resamplings are indicated for branches with >70% (gray dot) and >90% (black) support. Species of the genus *Dechloromonas* were used as the outgroup. The scale bar represents substitutions per nucleotide base.

When present, the 16S rRNA gene sequences from the MAGs were mapped against the MiDAS4 full-length amplicon sequence variant (ASV) database, which showed that the 16S rRNA gene was not able to resolve all genome-inferred species in the genus (Table 1 and Fig. 2). The 16S rRNA gene sequences extracted from the MAGs represented members of the MiDAS-defined species “*Ca.* Accumulibacter phosphatis” (4 MAGs), “*Ca.* Accumulibacter aalborgensis” (2 MAGs), and the *de novo* species midas_s_12920 (2 MAGs). This lack of taxonomic resolution could be explained by the more rapid evolution of the *ppk1* gene compared to that of the 16S rRNA gene, which is more conserved within the genus. The lack of resolution was also observed when analyzing 16S rRNA gene sequences across the ~500-bp amplicon sequence variants (V1 to V3 region) normally used for abundance estimation (Table 1). Among the most abundant “*Ca.* Accumulibacter” ASVs in the MiDAS4 global WWTP data set (Fig. S2), ASV402 was 100% identical to the V1 to V3 regions of both “*Ca.* Accumulibacter phosphatis” and “*Ca.* Accumulibacter delftensis” and ASV548 was identical to those of “*Ca.* Accumulibacter affinis,” “*Ca.* Accumulibacter proximus,” and “*Ca.* Accumulibacter propinquus,” complicating the interpretation of amplicon abundance studies based on 16S rRNA gene amplicon sequencing.

10.1128/msystems.00016-22.2 FIG S2 Mean read abundance of 10 most abundant amplicon sequence variants (ASVs) of different 16S-defined “*Ca*. Accumulibacter” species in global enhanced biological phosphorus removal (EBPR) wastewater treatment plants (WWTPs), sorted by country. Download FIG S2, PDF file, 0.2 MB. Copyright © 2022 Petriglieri et al. 2022 Petriglieri et al. https://creativecommons.org/licenses/by/4.0/ This content is distributed under the terms of the Creative Commons Attribution 4.0 International license.

The MiDAS4 database presented three more *de novo* species classified as “*Ca.* Accumulibacter,” as follows: midas_s_315, midas_s_168, and midas_s_3472, all based on full-length 16S rRNA genes. According to genome-based phylogeny (Fig. 1), midas_s_315 clustered with the isolate genome Propionivibrio dicarboxylicus (GenBank assembly number GCA_900099695) and with the “*Ca.* Propionivibrio aalborgensis” MAG (assembly number GCA_900089945), together with another novel species with the provisional name midas_s_421. We propose the name “*Candidatus* Propionivibrio dominans” for midas_s_315. The species midas_s_168 was represented by two MAGs, which clustered outside both “*Ca.* Accumulibacter” and *Propionivibrio*, representing an undescribed genus and species for which we propose the name “*Candidatus* Proximibacter danicus.” Due to the lack of a representative MAG, no confident taxonomic assignment could be made for the species midas_s_3472, which was investigated *in situ* (see below) to examine its capacity to carry out canonical PAO metabolism. This imprecise naming may be accounted for by the naive taxonomic assignment of the automated 16S rRNA-based taxonomy assignments with AutoTax, which uses a strict species identity cutoff 98.7% (27) that is less suited for “*Ca.* Accumulibacter” due to the high conservation of the 16S rRNA gene within this genus.

### Geographic distribution of “*Ca.* Accumulibacter” populations in global WWTPs.

**FIG 3:**
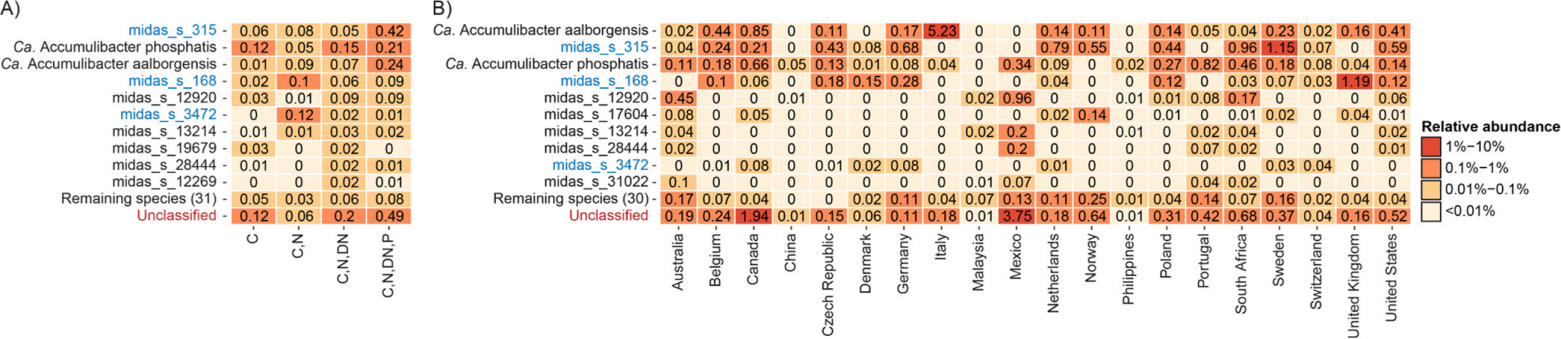
The 10 most abundant “*Ca*. Accumulibacter” species worldwide according to the MiDAS4 survey. (A) Mean relative abundance across different process configurations (C, carbon removal; N, nitrification; DN, denitrification; P, phosphorus removal). (B) Mean relative abundance in EBPR plants across different countries. Data originate from the global survey of microbial communities in WWTPs (29) and it is based on a V1 to V3 amplicon data set. Species marked in blue were wrongly classified as “*Ca*. Accumulibacter” according to the genome-based taxonomy.

Despite the lack of resolution of 16S rRNA gene amplicon studies for these lineages, the new MiDAS global reference database of full-length 16S rRNA gene sequences (29) ensures that amplicon analysis is still a powerful tool to analyze their geographical distribution. On a global scale, MiDAS-defined “*Ca.* Accumulibacter phosphatis,” “*Ca*. Accumulibacter aalborgensis,” and the *de novo* midas_s_12920, were the most abundant species within the “*Ca*. Accumulibacter” lineage (Fig. 3A and B). The distribution of “*Ca.* Accumulibacter” was strongly influenced by the activated sludge process configuration, with higher relative abundances of all species in EBPR plants (Fig. 3A). MiDAS-defined “*Ca*. Accumulibacter” species were present in several full-scale EBPR plants worldwide, with the highest relative abundances in Mexico (5.7%), Italy (5.5%), Canada (3.9%), and South Africa (2.5%) (Fig. 3B). “*Ca.* Propionivibrio dominans” (midas_s_315) was also observed in higher abundance in biological nutrient removal (BNR) plants with nitrogen and phosphorus removal (Fig. 3A), along with other *Propionivibrio* species (Fig. S3A), which are most likely favored by their glycogen-accumulating metabolism that can exploit resources in the alternating aerobic/anaerobic cycling typical of the EBPR design. The highest relative abundances were observed in Norway (2.6%), the Netherlands (1.7%), and Sweden (1.2%) (Fig. S3B). “*Ca.* Proximibacter danicus” was present also in WWTPs with simpler designs (Fig. S4A). The highest abundance was observed in the United Kingdom (1.2%), Germany (0.3%), and the Czech Republic (0.2%) (Fig. S4B).

10.1128/msystems.00016-22.3 FIG S3 Global average relative read abundance of top 5 MiDAS-defined Propionivibrio species. Download FIG S3, PDF file, 0.3 MB. Copyright © 2022 Petriglieri et al. 2022 Petriglieri et al. https://creativecommons.org/licenses/by/4.0/ This content is distributed under the terms of the Creative Commons Attribution 4.0 International license.

10.1128/msystems.00016-22.4 FIG S4 Global average relative read abundance of top 5 MiDAS-defined “*Candidatus* Proximibacter” species. Download FIG S4, PDF file, 0.2 MB. Copyright © 2022 Petriglieri et al. 2022 Petriglieri et al. https://creativecommons.org/licenses/by/4.0/ This content is distributed under the terms of the Creative Commons Attribution 4.0 International license.

The choice of primers has great importance for microbial profiling of activated sludge samples (37). According the MiDAS global study, “*Ca*. Accumulibacter” was equally well detected at the genus-level with primers targeting the V1 to V3 and V4 variable regions of the 16S rRNA gene (29). This is also in accordance with what was recently observed by Roy et al. (38). However, there may be clear differences in how good amplicons from different variable regions are at resolving the species-level diversity within specific genera (27). We therefore used the MiDAS4 reference database to determine the ecosystem-specific theoretical taxonomic resolution provided by amplicons targeting different variable regions of the 16S rRNA gene (Fig. S5A). This revealed that amplicons targeting the V1 to V3 region were better suited for resolving the species-level diversity within “*Ca*. Accumulibacter” than amplicons targeting the V4 region. This was also clear when we examined the global diversity of “*Ca*. Accumulibacter” species based on V4 amplicon data from the MiDAS global survey, which revealed that almost all ASVs were unclassified at the species level (Fig. S5B). These remarkable differences must be taken into consideration when comparing abundance estimates obtained with different primer sets, as well as if EBPR efficiency is evaluated using amplicon sequencing data.

10.1128/msystems.00016-22.5 FIG S5 Evaluation of the V1 to V3 and V4 primers for detection of “*Ca*. Accumulibacter” species. Download FIG S5, PDF file, 0.2 MB. Copyright © 2022 Petriglieri et al. 2022 Petriglieri et al. https://creativecommons.org/licenses/by/4.0/ This content is distributed under the terms of the Creative Commons Attribution 4.0 International license.

### *In situ* visualization of “*Ca.* Accumulibacter” and other related species.

**FIG 4:**
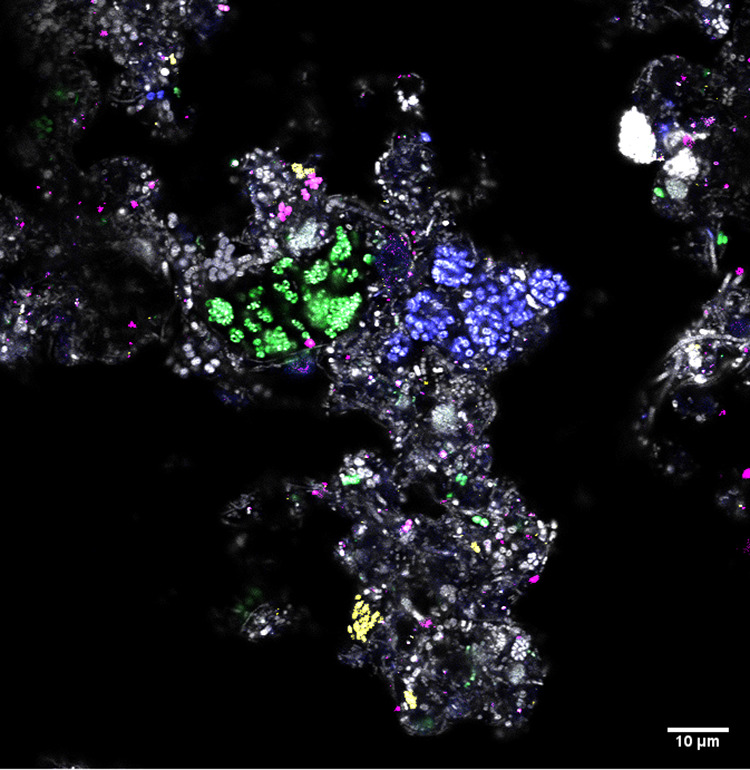
Multicolor FISH micrograph of different “*Ca*. Accumulibacter” species in full-scale activated sludge. “*Ca.* Accumulibacter proximus” (green) was targeted by the Acc471 probe. “*Ca*. Accumulibacter propinquus” (blue) was targeted by the Acc1011 probe. “*Ca*. Accumulibacter regalis” (magenta) was targeted by the Acc635 probe. “*Ca*. Accumulibacter delftensis” and “*Ca.* Accumulibacter aalborgensis” (yellow) were targeted by the Acc470 probe. All bacteria (gray) were targeted with the EUBmix probe. Bar, 10 μm.

Using the comprehensive set of ASV-resolved full-length 16S rRNA genes in the MiDAS4 database, we designed, when possible, species-specific FISH probes (Fig. 2 and Table S1). When targeting “*Ca*. Accumulibacter phosphatis,” “*Ca*. Accumulibacter affinis,” “*Ca*. Accumulibacter proximus,” “*Ca*. Accumulibacter propinquus,” “*Ca*. Accumulibacter iunctus,” “*Ca*. Accumulibacter similis,” and “*Ca*. Propionivibrio dominans,” the FISH probes hybridized with cocci of different diameters that were always arranged in small clusters inside the activated sludge floc (Table 2 and Fig. 4 and Fig. S6A to G). “*Ca*. Proximibacter danicus,” in contrast, was found as rod-shaped cells that were often attached to filamentous bacteria as epiphytic growth (Table 2 and Fig. S6H). A FISH probe designed to target the *de novo* species midas_s_3472 hybridized with low-abundance rod-shaped cells dispersed into the floc (Table 2 and Fig. S6I). The newly designed FISH probes were also applied to Danish and global activated sludge samples for quantitative FISH (Table S2). Compared to amplicon sequencing abundances, the FISH quantification provides an independent quantification based on biovolume of the specific “*Ca*. Accumulibacter” species. The species “*Ca.* Accumulibacter regalis,” “*Ca.* Accumulibacter affinis,” and “*Ca*. Accumulibacter proximus” were abundant (>1%) in samples with high read abundance of the 16S MiDAS-defined “*Ca.* Accumulibacter.” The MiDAS-defined “*Ca*. Accumulibacter aalborgensis” (which also covers “*Ca*. Accumulibacter delftensis”) was also present in high abundance in the samples analyzed. The FISH-based abundance estimates of “*Ca*. Accumulibacter iunctus” were significantly lower than expected based on amplicon sequencing (Table S2), perhaps due to their small biovolume per cell. “*Ca*. Propionivibrio dominans,” “*Ca*. Propionivibrio danicus,” and the *de novo* species midas_s_3472 were generally present as well, but in low abundances.

**TABLE 2:** Summary of features of different “*Ca.* Accumulibacter species”

Identifier	FISH probe	Morphology (diam × length [μm])	Storage polymer^*a*^		
			polyP	PHA	Glycogen
“*Ca.* Accumulibacter delftensis”	Acc470		+	+	+
“*Ca*. Accumulibacter regalis”	Acc635	Coccoid (0.4–0.6)	+	+	+
“*Ca.* Accumulibacter aalborgensis”	Acc470	Coccoid (0.7–0.9)	+	+	+
“*Ca*. Accumulibacter propinquus”	Acc1011	Coccoid (0.8–1.2)	+	+	+
“*Ca*. Accumulibacter affinis”	Acc471	Coccoid (0.5–0.7)	+	+	+
“*Ca.* Accumulibacter proximus”	Acc471	Coccoid (0.5–0.7)	+	+	+
“*Ca*. Accumulibacter iunctus”	Acc471_2	Coccoid (0.8–0.9)	+	+	+
“*Ca*. Accumulibacter similis”	Acc471_2	Coccoid (0.8–0.9)	+	+	+
“*Ca.* Proximibacter danicus”	Acc442	Rod-shaped (0.3–05 × 1–2)	−	+	−
“*Ca.* Propionivibrio dominans”	Acc213	Rod-shaped (0.5-0.6 × 0.9–1.1)	−	+	+
midas_s_3472	Acc441	Rod-shaped (0.3–0.4 × 0.8–1.2)	−	+	+

a Detected by Raman microspectroscopy.

10.1128/msystems.00016-22.6 FIG S6 Overlap of species-specific fluorescence *in situ* hybridization (FISH) probes for “*Ca*. Accumulibacter” and the widely applied probe PAO651. Download FIG S6, PDF file, 0.3 MB. Copyright © 2022 Petriglieri et al. 2022 Petriglieri et al. https://creativecommons.org/licenses/by/4.0/ This content is distributed under the terms of the Creative Commons Attribution 4.0 International license.

10.1128/msystems.00016-22.8 TABLE S1 Summary of fluorescence *in situ* hybridization (FISH) probes used in this study. Download Table S1, DOCX file, 0.02 MB. Copyright © 2022 Petriglieri et al. 2022 Petriglieri et al. https://creativecommons.org/licenses/by/4.0/ This content is distributed under the terms of the Creative Commons Attribution 4.0 International license.

10.1128/msystems.00016-22.9 TABLE S2 Comparison of quantitative FISH (qFISH) and 16S rRNA amplicon results (V1 to V3). Download Table S2, DOCX file, 0.02 MB. Copyright © 2022 Petriglieri et al. 2022 Petriglieri et al. https://creativecommons.org/licenses/by/4.0/ This content is distributed under the terms of the Creative Commons Attribution 4.0 International license.

The specificity of the widely applied PAOmix probe set (4) was evaluated *in silico* (Fig. 2) and *in situ* (Fig. S6). It showed lower specificity than expected, targeting various *Propionivibrio* spp., including “*Ca*. Propionivibrio dominans.” Similarly, we tested *in silico* (Fig. 2) the coverage and specificity of the “type” FISH probes Acc-I-444 and Acc-II-444 (19). While Acc-I-444 targets several 16S rRNA gene sequences belonging to “*Ca*. Accumulibacter aalborgensis” and “*Ca*. Propionivibrio aalborgensis,” Acc-II-444 showed more specific coverage of the MiDAS-defined “*Ca*. Accumulibacter phosphatis” cluster. The unspecific binding of the PAOmix and the “type” probes could explain why previous studies observed two populations of “*Ca*. Accumulibacter” with different morphologies (coccoid and rod shaped) and wrongly concluded that there was a morphological difference between the two *ppk1*-defined types (16, 19).

### Potential for polyP, glycogen, and PHA accumulation and *in situ* validation.

**FIG 5:**
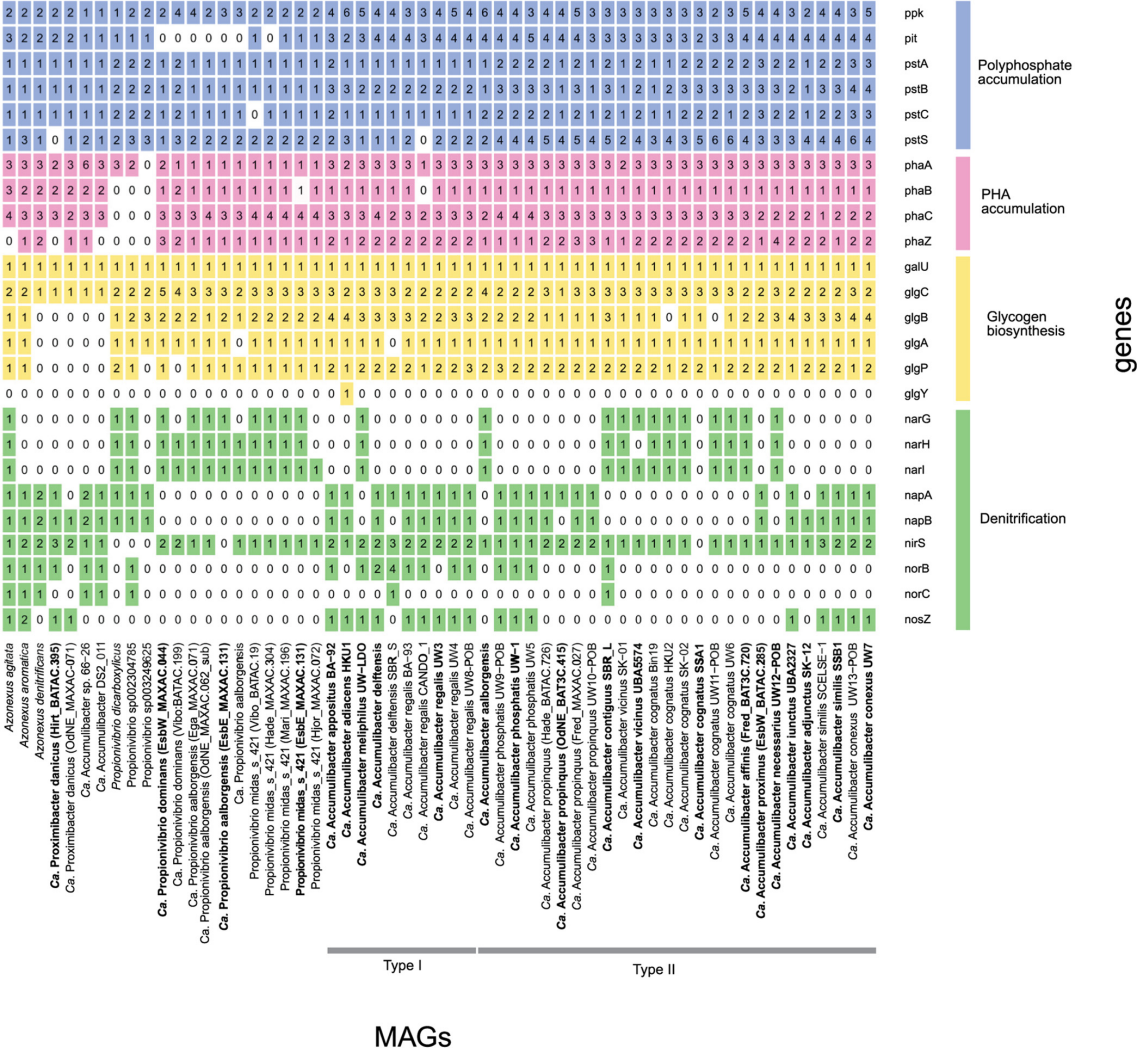
PAO metabolism-related functional potential of the “*Ca*. Accumulibacter” MAGs and closest relatives. The gene list follows the progression in the text. For the full list of gene names and associated KO numbers, see Data File S2 (available at https://doi.org/10.6084/m9.figshare.17306828.v1). The MAGs and genomes are ordered as in the genome tree in Fig. 1.

Genome mining for genes and pathways related to the PAO metabolism of the MAGs based on Kyoto Encyclopedia of Genes and Genomes (KEGG) orthology was performed to confirm the potential for PAO metabolism. All of the “*Ca*. Accumulibacter” sp. genomes encoded essential genes for polyphosphate accumulation and storage, such as the low-affinity phosphate transporter (*pit*) and the high-affinity phosphate transporter (*pstSCAB*) (Fig. 5; see also Data File S2 at https://doi.org/10.6084/m9.figshare.17306828.v1). The MAGs also encoded full potential for glycogen and PHA accumulation, typical of the PAO phenotype (Fig. 5; see also Data File S2 at https://doi.org/10.6084/m9.figshare.17306828.v1). These metabolic predictions were further confirmed *in situ* by the presence of intracellular polyP, PHA, and glycogen by FISH-Raman analysis (Table 2 and Fig. S7). The MAGs belonging to “*Ca*. Propionivibrio dominans” encoded the full potential for PHA and glycogen accumulation, but not for polyP storage, previously observed for “*Ca*. Propionivibrio aalborgensis” (Fig. 5 and reference 26). These metabolic predictions were also confirmed by FISH-Raman analysis (Fig. S7), which showed a similar intracellular profile for the *de novo* species midas_s_3472, representing additional evidence to support the different taxonomic classification of these lineages. Similarly, the MAGs belonging to “*Ca*. Proximibacter danicus” encoded the potential for polyP and PHA accumulation, but only the latter was detected *in situ*.

10.1128/msystems.00016-22.7 FIG S7 Raman spectra of “*Ca*. Accumulibacter aalborgensis” and “*Ca*. Propionivibrio dominans” highlighting the differences between the two genera in storage polymer content. Download FIG S7, PDF file, 0.04 MB. Copyright © 2022 Petriglieri et al. 2022 Petriglieri et al. https://creativecommons.org/licenses/by/4.0/ This content is distributed under the terms of the Creative Commons Attribution 4.0 International license.

Differences in nitrate and nitrite reduction potential have often been suggested as a determining factor for niche and type differentiation, representing one of the most controversial (and arguably consequential) features of the “*Ca*. Accumulibacter” physiology (7, 19, 39, 40). Therefore, we also analyzed the distribution of genes involved in the denitrification process in “*Ca.* Accumulibacter” and related species (Fig. 5). Genes encoding the respiratory nitrate reductase NarGHI were detected in “*Ca*. Accumulibacter meliphilus” (1 MAG), “*Ca*. Accumulibacter aalborgensis” (1 MAG), “*Ca*. Accumulibacter contiguus” (1 MAG), “*Ca*. Accumulibacter vicinus” (2 MAGs), “*Ca*. Accumulibacter cognatus” (5/6 MAGs), “*Ca*. Accumulibacter affinis” (1 MAG), and “*Ca.* Accumulibacter necessarius” (1 MAG), while the other “*Ca.* Accumulibacter” MAGs carried the genes coding for the periplasmic nitrate reductase NapAB. Although both enzymes can carry out nitrate reduction, the presence of the NarGHI enzyme complex was previously shown to be essential for anoxic phosphorus uptake using nitrate (39, 40). Its absence in the majority of the MAGs may indicate an inability to generate sufficient proton motive force to support respiration coupled to phosphorus uptake. On the contrary, the potential for nitrite reduction was more widespread, with *nirS* identified in all “*Ca*. Accumulibacter” MAGs except that of “*Ca*. Accumulibacter cognatus.” Nitric oxide reductase (*norBC*) was present only in 2 MAGs, representing “*Ca.* Accumulibacter delftensis” SBR_S and “*Ca.* Accumulibacter” contiguous SBR_L, while nitrous oxide reductase (*nosZ*) was encoded by “*Ca*. Accumulibacter appositus” (1 MAG), “*Ca.* Accumulibacter adiacens” (1 MAG), “*Ca.* Accumulibacter meliphilus” (1 MAG), “*Ca.* Accumulibacter delftensis” (1/2 MAGs), “*Ca.* Accumulibacter regalis” (5/6 MAGs), “*Ca.* Accumulibacter iunctus” (1 MAG), “*Ca*. Accumulibacter similis” (2/2 MAG), and “*Ca.* Accumulibacter conexus” (2/2 MAGs). Our metabolic predictions for “*Ca*. Accumulibacter delftensis” slightly differed from those of Rubio-Rincon et al. (40), where they identified genes encoding the periplasmic nitrate reductase (*nap*) and a full set of nitrite (*nir*), nitric oxide (*nor*), and nitrous oxide (*nos*) reductases. Manual inspection of the “*Ca*. Accumulibacter delftensis” genome using the MicroScope platform (41) revealed the presence of genes coding for full reduction of nitrite to nitrogen gas. The distribution of the genes analyzed in this study did not show any evidence supporting the hypothesis of a physiological distinction between *ppk1*-defined type I and type II within the genus “*Ca*. Accumulibacter.” On the contrary, the specific set of genes involved in denitrification seems to be species dependent.

Similarly, “*Ca*. Propionivibrio dominans” and “*Ca*. Proximibacter danicus” genomes carried genes for nitrate and nitrite reduction and also differed in their nitrate reductase gene (*narGHI* versus *napAB*, respectively). The *nirS* gene was identified in both. “*Ca*. Proximibacter danicus” MAGs encoded also nitrous oxide reductase (NosZ). These metabolic differences across the genus “*Ca*. Accumulibacter” and the other related genera/species could explain why these different taxa could coexist in the same ecosystems and contribute to overall phosphorus and nitrogen removal. However, experimental validation of these metabolic predictions is needed to confirm their metabolic abilities and determine their contribution to the full-scale EBPR process.

### Conclusions and perspectives.

Here, we provide a long-needed reassessment of the phylogeny of the genus “*Ca.* Accumulibacter,” using a comparison of genome, *ppk1*, and 16S rRNA gene approaches, and identified 18 novel species, for which we propose “*Candidatus*” names. We verified that the 16S rRNA gene is not able to resolve the phylogeny of these lineages and should be applied with caution in amplicon studies. The *ppk1* gene is confirmed as the best choice for this purpose and offers a higher resolution in distinguishing the different species. However, despite being 16S rRNA gene-based, a global survey such as the MiDAS4 can offer valuable insights to investigate the geographical distribution and major drivers of environmental filtering. As expected, “*Ca*. Accumulibacter” taxa had higher relative abundance in WWTPs performing biological phosphorus removal, indicating the process design as a major factor influencing their abundance. We also investigated the influence of the primer set chosen for the amplicon analysis and showed that despite being incapable of distinguishing all of the different species, the V1 to V3 primer set was more suitable than the V4 set, which was unable to provide species-level resolution.

Finally, the species-specific FISH probes designed in this study, applied in combination with Raman microspectroscopy, confirmed the presence of the typical PAO storage polymers predicted by metabolic annotation of the MAGs. The MAGs were investigated for the distribution of genes encoding the denitrification pathway related to one of the most controversial physiological traits of the “*Ca.* Accumulibacter” clades. The annotation revealed fine-scale differences in the stepwise nitrogen-species reduction pathways, giving some insights into the niche differentiation of these lineages. Future experiments, for example, using transcriptomics or activity-based studies with stable isotope-labeled compounds and FISH-Raman, could help confirm the metabolic abilities of the different species and explain how they can coexist in the same ecosystem and contribute to overall phosphorus and nitrogen removal.

### Etymology.

Proposed etymologies and protologues for the novel proposed species are provided in Text S1 in the supplemental material.

10.1128/msystems.00016-22.10 TEXT S1 Taxonomic proposal and protologue tables. Download Text S1, DOCX file, 0.06 MB. Copyright © 2022 Petriglieri et al. 2022 Petriglieri et al. https://creativecommons.org/licenses/by/4.0/ This content is distributed under the terms of the Creative Commons Attribution 4.0 International license.

## MATERIALS AND METHODS

### Sampling and fixation.

Sampling of activated sludge was carried out within the Danish MiDAS project (28, 42) and the global MiDAS project (29). In short, fresh biomass samples from the aeration tank from various WWTPs were collected and either sent to Aalborg University (Danish MiDAS) or preserved in RNAlater and shipped to Aalborg University with cooling elements (Global MiDAS). Upon arrival, all samples were subsampled and stored at −20°C for sequencing workflows, then fixed for FISH with 50% ethanol (final volume) or 4% PFA (final volume) as previously described (43).

### DNA extraction and community profiling using 16S rRNA gene amplicon sequencing.

DNA extraction, sample preparation, and amplicon sequencing were performed as described by Nierychlo et al. (28) and Dueholm et al. (29). V1 to V3 16S rRNA gene regions were amplified using the 27F (5′-AGAGTTTGATCCTGGCTCAG-3′) (44) and 534R (5′-ATTACCGCGGCTGCTGG-3′) (45) primers, and the resulting amplicons were used in all the analyses. The V4 16S rRNA gene region was amplified using the 515F (5′-GTGYCAGCMGCCGCGGTAA-3′) (46) and 806R (5′-GGACTACNVGGGTWTCTAAT-3′) (47) primers for comparison with the previous data set. Data were analyzed using R (version 3.5.2) (48) through RStudio software (49) and visualized using ampvis2 (version 2.7.5) (50) and ggplot2 (51). Theoretical evaluation of the taxonomic resolution provided by different variable regions of the 16S rRNA gene was determined by extracting *in silico* ASV corresponding to each variable region from references in the MiDAS4 database, classifying them with the full database, and calculating the percentages of correct and wrong classifications using the MiDAS4 taxonomy as the ground truth.

### Phylogenomic analysis and MAG annotation.

MAGs identified as potential “*Ca*. Accumulibacter” or *Propionivibrio*, a close relative to the former, were obtained from a set of 1,083 high-quality (HQ) MAGs recovered from Danish WWTPs (25). Identification was based on the GTDB-Tk v1.4.1 (30) taxonomy classification of the MAGs and mapping of extracted 16S rRNA genes against the MiDAS 3 database (25, 28) using “usearch -global.” MAGs with either genome taxonomy or 16S rRNA gene classification as “*Ca*. Accumulibacter” or *Propionivibrio* were selected for further investigation. These genomes were added to a collection of publicly available HQ MAGs (12, 24, 30–33) selected based on quality standards proposed by Bowers et al. (52) (completeness and contamination of >90% and <5%, respectively) (for accession numbers and leaf names, see Data File S1 at https://doi.org/10.6084/m9.figshare.17306771.v1). The IA-UW2 strain assembled by Flowers et al. (10) was renamed UW3, after removing a prophage contig. Concatenated and trimmed alignments of 120 single-copy marker gene proteins were created using GTDB-Tk “classify.” The multiple sequence alignments included the selection of the MAGs mentioned above, as well as three *Azonexus* (formerly *Dechloromonas*) isolates (GenBank assembly numbers IMG taxon_id 637000088, GCA_000519045.1, and GCA_001551835.1) used as outgroups to create a rooted tree. The alignment was used as input for IQ-TREE v2 (53) to create a genome tree using the WAG+G model with 100 bootstrap iterations. dRep v2.3.2 (54) “-comp 50 -con 10 -sa 0.95” was used to dereplicate the genomes at 95% ANI to indicate the species representatives in the genome tree. Pairwise ANI was calculated for all “*Ca.* Accumulibacter” genomes using fastANI (55) and ordered in the same order as the phylogenetic tree in Fig. 1. MAGs and genomes were annotated as described previously (56). Briefly, EnrichM v0.5.0 (https://github.com/geronimp/enrichM) “annotate” was used to annotate the genes with KEGG (57) Orthology (KO) numbers using a DIAMOND v0.9.22 (58) BLAST search against the KO-annotated UniRef100 database (EnrichM database v10). EnrichM “classify” with “-cutoff 1” was then used to determine the presence of 100% complete KEGG modules. The output used in this study are presented in Data File S2 (available at https://doi.org/10.6084/m9.figshare.17306828.v1). Additionally, the MAGs were uploaded to the MicroScope Microbial Genome Annotation & Analysis Platform (MAGE) (41) in order to cross-validate KO annotations found using EnrichM.

### *ppk1* phylogenetic analysis.

The *ppk1* gene sequences were sourced from the database file from McDaniel et al. (32) (https://github.com/elizabethmcd/ppk1_Database). Additional *ppk1* sequences from MAGs in the Genome Taxonomy Database (GTDB) and *Propionivibrio* and *Azonexus* MAGs were sourced from the genomes (Data File S3, available at https://doi.org/10.6084/m9.figshare.17306849.v1). Prokka v1.14 (59) was used to call and annotate the genes within the genomes, enabling identification of the polyphosphate kinase gene (*ppk1*), which was extracted using Fxtract v2.3 (https://github.com/ctSkennerton/fxtract) and added to the *ppk1* database file. The *ppk1* gene sequences were aligned using MAFFT v7.47 (60) with the “mafft -auto” command. The alignment was inputted into IQ-TREE v2 with Model Finder enabled using “-m MFP.” The GTR+F+I+G4 model was selected by Model Finder, and a phylogenetic maximum likelihood tree was created with 100 bootstrap iterations. ARB v6.0.3 (61) was used to visualize the trees and set the root based on the outgroup sequences. The trees were exported to iTOL v6 (62), enabling the nodes to be matched up as much as possible for presentation in Fig. 1. Final esthetic processing was done in Inkscape v1.0.2.

### 16S rRNA gene phylogenetic analysis, FISH probe design, and evaluation.

Phylogenetic analysis of 16S rRNA gene sequences and design of FISH probes were performed using ARB software v.6.0.6 (61). 16S rRNA gene sequences were extracted from the MAG gene files created by Prokka (.ffn files) using Fxtract and were also retrieved from the MiDAS4 database (29) and a publicly available set (3). A phylogenetic tree was calculated based on comparative analysis of aligned 16S rRNA gene sequences using the maximum likelihood method and a 1,000-replicate bootstrap analysis. Coverage and specificity of the FISH probes were evaluated and validated *in silico* with the MathFISH software for hybridization efficiencies of target and potentially weak nontarget matches (63). When needed, unlabeled competitors were designed. All probes were purchased from Biomers (Ulm, Germany), labeled with cyanine 3 (Cy3), cyanine 5 (Cy5), 6-Carboxyfluorescein (6-FAM), Atto 532, Atto 565, Atto 594, and Atto 633 fluorochromes.

### FISH, quantitative FISH, and Raman microspectroscopy.

FISH was performed as previously described (43). Optimal formamide concentration for FISH probes was determined after performing hybridization at different formamide concentrations in the range of 0 to 70% (with 5% increments). The intensity of at least 50 cells was measured using ImageJ (64) software. Optimal hybridization conditions are described in Table S1 in the supplemental material. EUBmix (65, 66) was used to target all bacteria, and NON-EUB (67) was used as a negative control for sequence-independent probe binding. Quantitative FISH (qFISH) biovolume fractions of individual genera were calculated as a percentage area of the total biovolume, hybridizing with both EUBmix probes and a specific probe. qFISH analyses, performed using the Daime image analysis software (68), were based on 30 fields of view taken at ×63 magnification. Microscopic analysis was performed with an Axioskop epifluorescence microscope (Carl Zeiss, Germany) equipped with a Leica DFC7000 T charge-coupled device (CCD) camera or a white light laser confocal microscope (TCS SP8 X; Leica). Multicolor FISH was performed as described by Lukumbuzya et al. (69). Raman microspectroscopy combined with FISH was used to detect intracellular storage polymers (polyP, PHA, and glycogen) in probe-defined species and was performed as previously described (70).

### Data availability.

All supplemental data files used in this study are available at https://figshare.com/projects/Re-evaluation_of_the_phylogenetic_diversity_and_global_distribution_of_the_genus_Candidatus_Accumulibacter_-_supplementary_files/129092.
