# Supplementary figures and images for: Reevaluation of the Phylogenetic Diversity and Global Distribution of the Genus “Candidatus Accumulibacter”
Source: mSystems. 2022 Apr 25;7(3):e00016-22. doi: 10.1128/msystems.00016-22 (PMC9238405; doi:10.1128/msystems.00016-22)

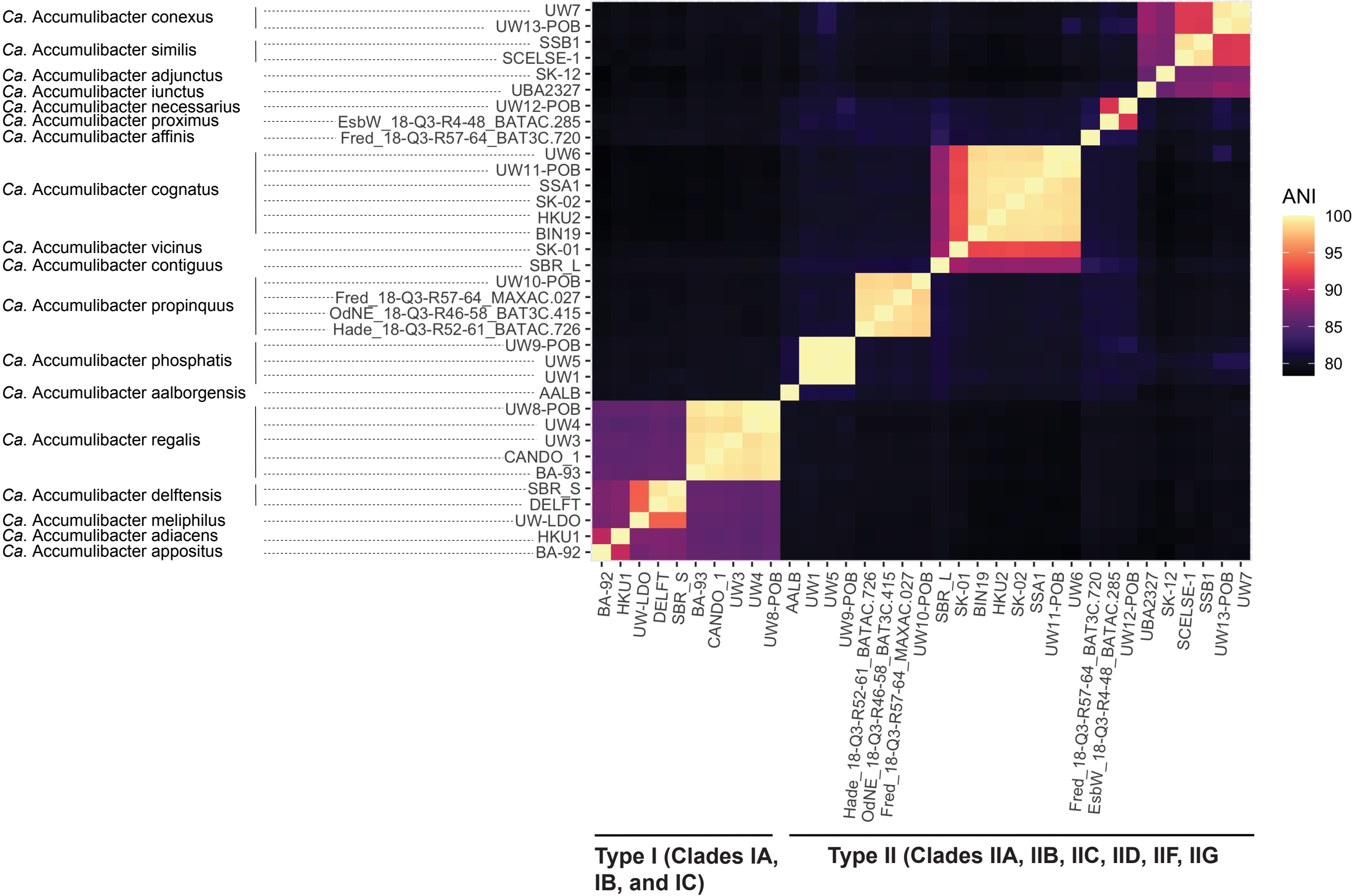

Supplement: FIG S1 [file msystems.00016-22-s0001.pdf]

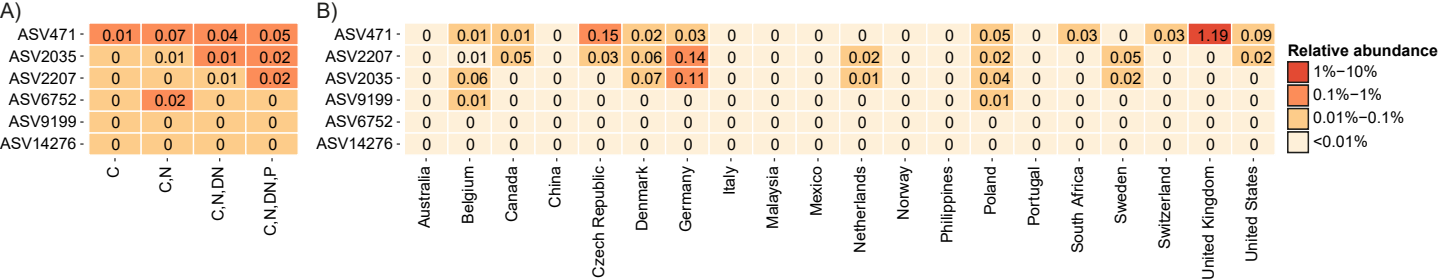

Supplement: FIG S4 [file msystems.00016-22-s0004.pdf]

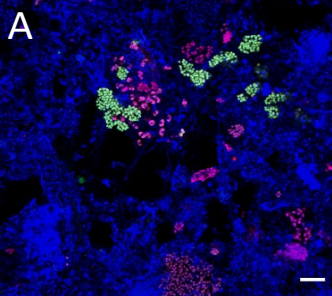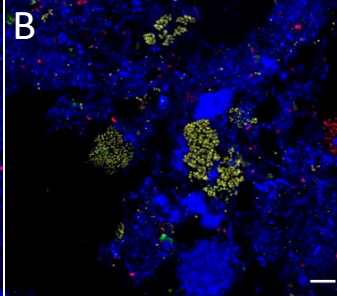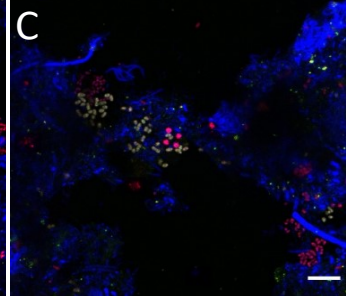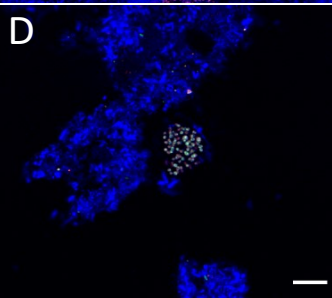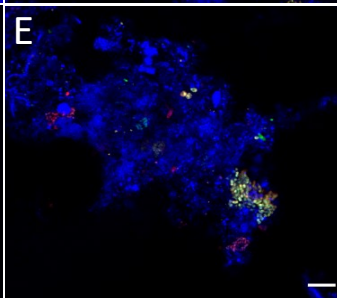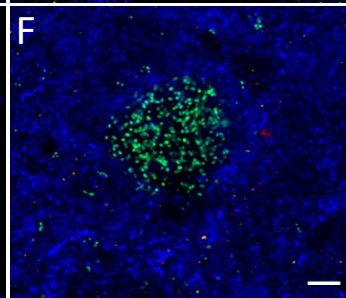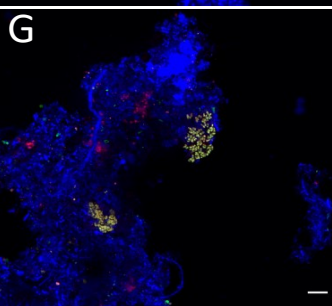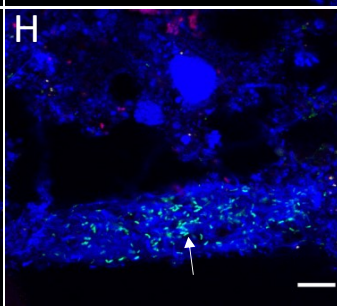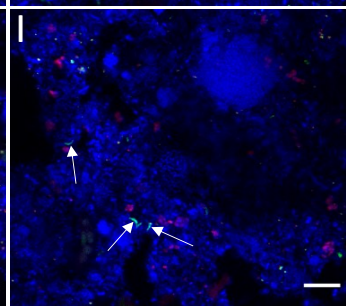

Supplement: FIG S6 [file msystems.00016-22-s0006.pdf]

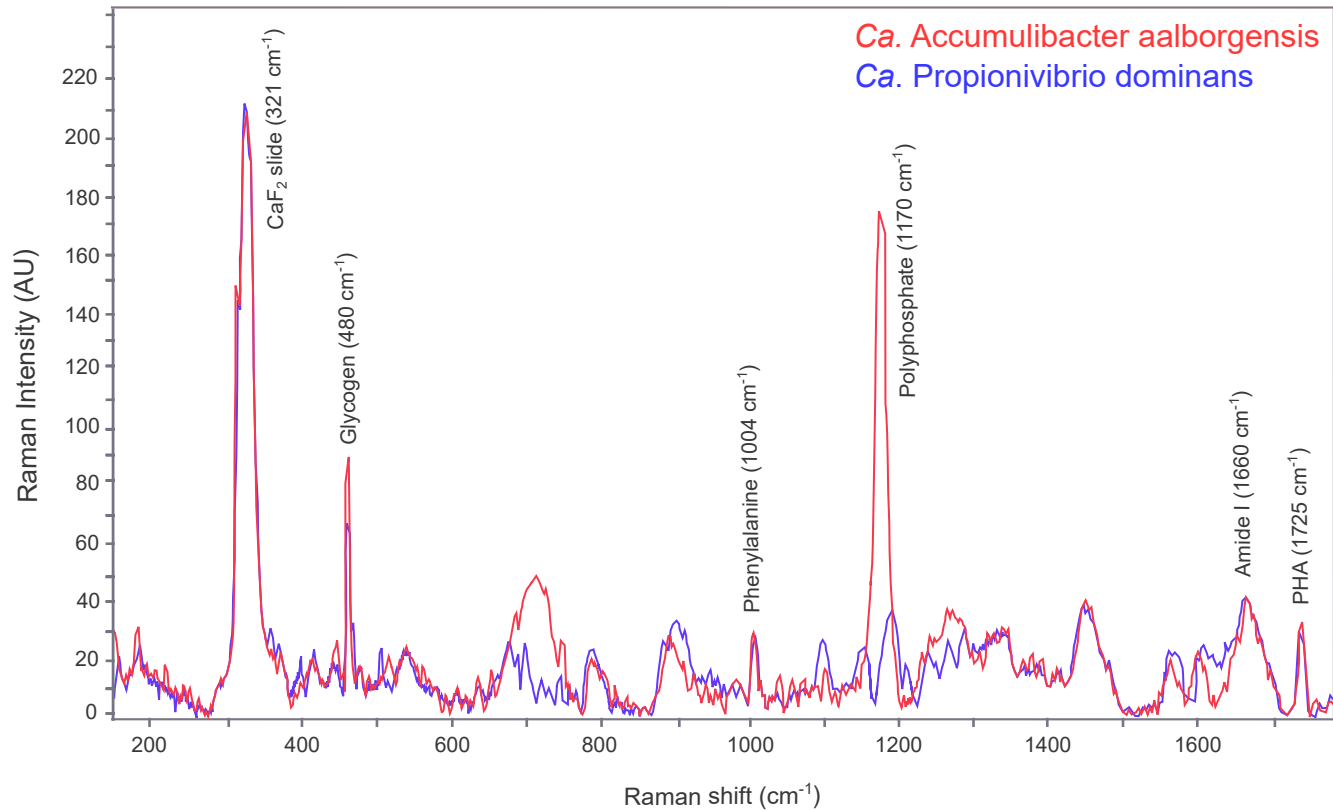

Supplement: FIG S7 [file msystems.00016-22-s0007.pdf]
