# Supplementary material for: Reevaluation of the Phylogenetic Diversity and Global Distribution of the Genus “Candidatus Accumulibacter”
Source: mSystems. 2022 Apr 25;7(3):e00016-22. doi: 10.1128/msystems.00016-22 (PMC9238405; doi:10.1128/msystems.00016-22)
Supplement: FIG S2 [file msystems.00016-22-s0002.pdf]

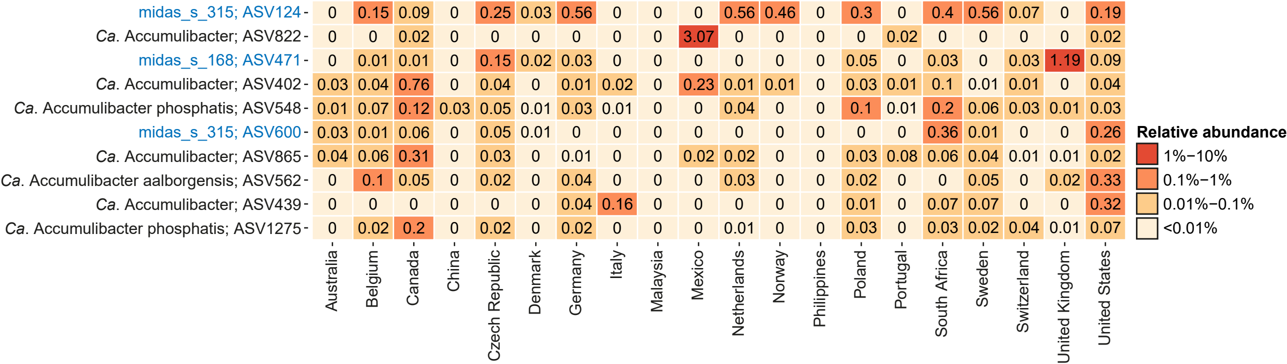
midas_s_315; ASV124 0 0.15 0.09 0 0.25 0.03 0.56 0 0 0 0.56 0.46 0 0.3 0 0.4 0.56 0.07 0 0.19
Ca. Accumulibacter; ASV822 0 0 0.02 0 0 0 0 0 0 3.07 0 0 0 0 0.02 0 0 0 0 0.02
midas_s_168; ASV471 0 0.01 0.01 0 0.15 0.02 0.03 0 0 0 0 0 0 0.05 0 0.03 0 0.03 1.19 0.09
Ca. Accumulibacter; ASV402 0.03 0.04 0.76 0 0.04 0 0.01 0.02 0 0.23 0.01 0.01 0 0.03 0.01 0.1 0.01 0.01 0 0.04
Ca. Accumulibacter phosphatis; ASV548 0.01 0.07 0.12 0.03 0.05 0.01 0.03 0.01 0 0 0.04 0 0 0.1 0.01 0.2 0.06 0.03 0.01 0.03
midas_s_315; ASV600 0.03 0.01 0.06 0 0.05 0.01 0 0 0 0 0 0 0 0 0 0.36 0.01 0 0 0.26
Ca. Accumulibacter; ASV865 0.04 0.06 0.31 0 0.03 0 0.01 0 0 0.02 0.02 0 0 0.03 0.08 0.06 0.04 0.01 0.01 0.02
Ca. Accumulibacter aalborgensis; ASV562 0 0.1 0.05 0 0.02 0 0.04 0 0 0 0.03 0 0 0.02 0 0 0.05 0 0.02 0.33
Ca. Accumulibacter; ASV439 0 0 0 0 0 0 0.04 0.16 0 0 0 0 0 0.01 0 0.07 0.07 0 0 0.32
Ca. Accumulibacter phosphatis; ASV1275 0 0.02 0.2 0 0.02 0 0.02 0 0 0 0.01 0 0 0.03 0 0.03 0.02 0.04 0.01 0.07
Australia
Belgium
Canada
China
Czech Republic
Denmark
Germany
Italy
Malaysia
Mexico
Netherlands
Norway
Philippines
Poland
Portugal
South Africa
Sweden
Switzerland
United Kingdom
United States
Relative abundance
1%−10%
0.1%−1%
0.01%−0.1%
<0.01%
