# Supplementary material for: Reevaluation of the Phylogenetic Diversity and Global Distribution of the Genus “Candidatus Accumulibacter”
Source: mSystems. 2022 Apr 25;7(3):e00016-22. doi: 10.1128/msystems.00016-22 (PMC9238405; doi:10.1128/msystems.00016-22)
Supplement: FIG S3 [file msystems.00016-22-s0003.pdf]

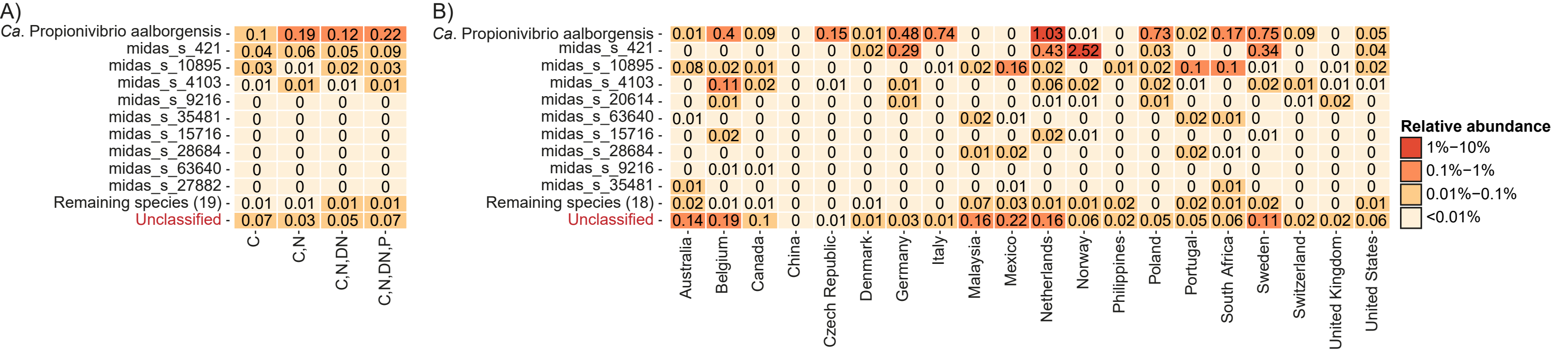
A)
Ca. Propionivibrio aalborgensis 0.1 0.19 0.12 0.22
midas_s_421 0.04 0.06 0.05 0.09
midas_s_10895 0.03 0.01 0.02 0.03
midas_s_4103 0.01 0.01 0.01 0.01
midas_s_9216 0 0 0 0
midas_s_35481 0 0 0 0
midas_s_15716 0 0 0 0
midas_s_28684 0 0 0 0
midas_s_63640 0 0 0 0
midas_s_27882 0 0 0 0
Remaining species (19) 0.01 0.01 0.01 0.01
Unclassified 0.07 0.03 0.05 0.07
C
C,N
C,N,DN
C,N,DN,P
B)
Ca. Propionivibrio aalborgensis 0.01 0.4 0.09 0 0.15 0.01 0.48 0.74 0 0 1.03 0.01 0 0.73 0.02 0.17 0.75 0.09 0 0.05
midas_s_421 0 0 0 0 0 0.02 0.29 0 0 0 0.43 2.52 0 0.03 0 0 0.34 0 0 0.04
midas_s_10895 0.08 0.02 0.01 0 0 0 0 0.01 0.02 0.16 0.02 0 0.01 0.02 0.1 0.1 0.01 0 0.01 0.02
midas_s_4103 0 0.11 0.02 0 0.01 0 0.01 0 0 0 0.06 0.02 0 0.02 0.01 0 0.02 0.01 0.01 0.01
midas_s_20614 0 0.01 0 0 0 0 0.01 0 0 0 0.01 0.01 0 0.01 0 0 0 0.01 0.02 0
midas_s_63640 0.01 0 0 0 0 0 0 0 0.02 0.01 0 0 0 0 0.02 0.01 0 0 0 0
midas_s_15716 0 0.02 0 0 0 0 0 0 0 0 0.02 0.01 0 0 0 0 0.01 0 0 0
midas_s_28684 0 0 0 0 0 0 0 0 0.01 0.02 0 0 0 0 0.02 0.01 0 0 0 0
midas_s_9216 0 0.01 0.01 0 0 0 0 0 0 0 0 0 0 0 0 0 0 0 0 0
midas_s_35481 0.01 0 0 0 0 0 0 0 0 0.01 0 0 0 0 0 0.01 0 0 0 0
Remaining species (18) 0.02 0.01 0.01 0 0 0.01 0 0 0.07 0.03 0.01 0.01 0.02 0 0.02 0.01 0.02 0 0 0.01
Unclassified 0.14 0.19 0.1 0 0.01 0.01 0.03 0.01 0.16 0.22 0.16 0.06 0.02 0.05 0.05 0.06 0.11 0.02 0.02 0.06
Australia
Belgium
Canada
China
Czech Republic
Denmark
Germany
Italy
Malaysia
Mexico
Netherlands
Norway
Philippines
Poland
Portugal
South Africa
Sweden
Switzerland
United Kingdom
United States
Relative abundance
1%−10%
0.1%−1%
0.01%−0.1%
<0.01%
