# Supplementary material for: Reevaluation of the Phylogenetic Diversity and Global Distribution of the Genus “Candidatus Accumulibacter”
Source: mSystems. 2022 Apr 25;7(3):e00016-22. doi: 10.1128/msystems.00016-22 (PMC9238405; doi:10.1128/msystems.00016-22)
Supplement: FIG S5 [file msystems.00016-22-s0005.pdf]

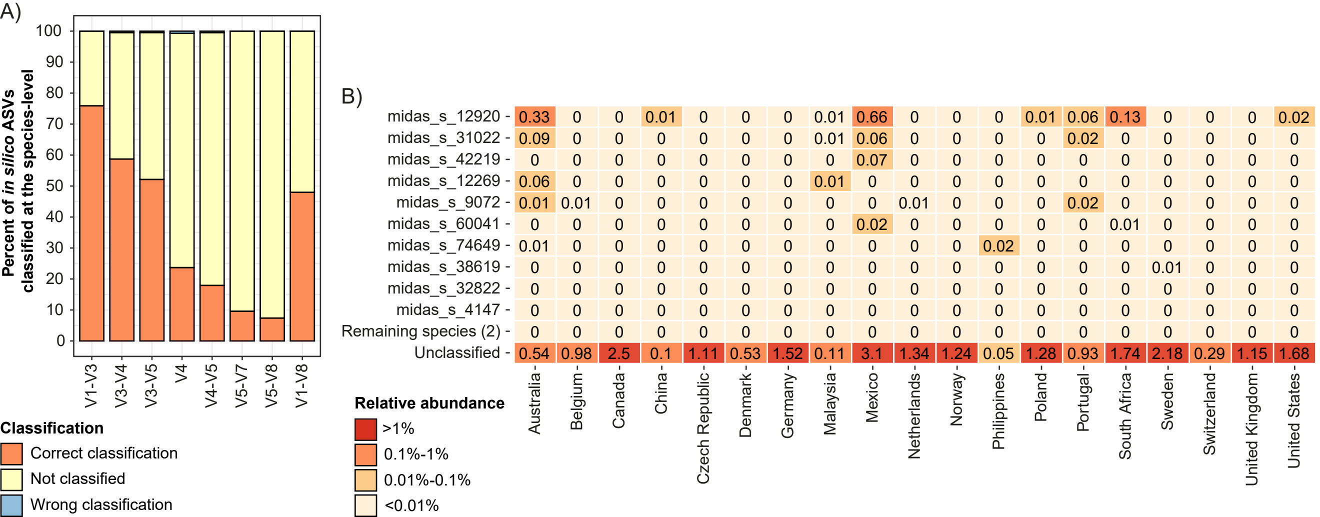

A)
Percent of in silico ASVs classified at the species-level
100
90
80
70
60
50
40
30
20
10
0
V1-V3
V3-V4
V3-V5
V4
V4-V5
V5-V7
V5-V8
V1-V8
Classification
Correct classification
Not classified
Wrong classification
B)
midas_s_12920 - 0.33 0 0 0.01 0 0 0 0.01 0.66 0 0 0 0.01 0.06 0.13 0 0 0 0.02
midas_s_31022 - 0.09 0 0 0 0 0 0 0.01 0.06 0 0 0 0 0.02 0 0 0 0 0
midas_s_42219 - 0 0 0 0 0 0 0 0 0.07 0 0 0 0 0 0 0 0 0 0
midas_s_12269 - 0.06 0 0 0 0 0 0 0.01 0 0 0 0 0 0 0 0 0 0 0
midas_s_9072 - 0.01 0.01 0 0 0 0 0 0 0 0.01 0 0 0 0.02 0 0 0 0 0
midas_s_60041 - 0 0 0 0 0 0 0 0 0.02 0 0 0 0 0 0.01 0 0 0 0
midas_s_74649 - 0.01 0 0 0 0 0 0 0 0 0 0 0.02 0 0 0 0 0 0 0
midas_s_38619 - 0 0 0 0 0 0 0 0 0 0 0 0 0 0 0 0.01 0 0 0
midas_s_32822 - 0 0 0 0 0 0 0 0 0 0 0 0 0 0 0 0 0 0 0
midas_s_4147 - 0 0 0 0 0 0 0 0 0 0 0 0 0 0 0 0 0 0 0
Remaining species (2) - 0 0 0 0 0 0 0 0 0 0 0 0 0 0 0 0 0 0 0
Unclassified - 0.54 0.98 2.5 0.1 1.11 0.53 1.52 0.11 3.1 1.34 1.24 0.05 1.28 0.93 1.74 2.18 0.29 1.15 1.68
Australia
Belgium
Canada
China
Czech Republic
Denmark
Germany
Malaysia
Mexico
Netherlands
Norway
Philippines
Poland
Portugal
South Africa
Sweden
Switzerland
United Kingdom
United States
Relative abundance
>1%
0.1%-1%
0.01%-0.1%
<0.01%
