# Supplementary material for: Reevaluation of the Phylogenetic Diversity and Global Distribution of the Genus “Candidatus Accumulibacter”
Source: mSystems. 2022 Apr 25;7(3):e00016-22. doi: 10.1128/msystems.00016-22 (PMC9238405; doi:10.1128/msystems.00016-22)
Supplement: TABLE S1 [file msystems.00016-22-s0008.docx]

**Table S1. Summary table of the FISH probes used in this study.**

| **Probe** | ***E. coli* pos.** | **Target group** | **Coverage*** | **Non-target hits** | **Sequence (5’-3’)** | **[FA]%**** | **Reference** |
| --- | --- | --- | --- | --- | --- | --- | --- |
| **PAO651** | **651–668** | **PAO cluster** | **44/467** |  | **CCC TCT GCC AAA CTC CAG** | **35** | **(1)** |
| **Acc469** | **469-493** | ***Ca.* Accumulibacter proximus** | **2/3** | **0** | **CCA GGT ACC GTC ATC TAC ACA GGC** | **30** | **This study** |
| Acc469_C1 | 469-493 | Competitor for Acc469 | N/A | N/A | CCA GGT ACC GTC ATC TAC ACA GGG | N/A | This study |
| Acc469_C2 | 469-493 | Competitor for Acc469 | N/A | N/A | CTA GGT ACC GTC ATC TAC ACA GGC | N/A | This study |
| Acc469_C3 | 469-493 | Competitor for Acc469 | N/A | N/A | CWA GGT ACC GTC ATC TAC ACA GGG | N/A | This study |
| Acc469_C4 | 469-493 | Competitor for Acc469 | N/A | N/A | TCA GGT ACC GTC ATC TAC ACA GGG | N/A | This study |
| **Acc471** | **471-495** | ***Ca.* Accumulibacter affinis and proximus** | **39/61** | **1** | **CTC CAG GTA CCG TCA TCT ACA CAG** | **40** | **This study** |
| Acc471_C1 | 471-495 | Competitor for Acc471 | N/A | N/A | CTC CGG GTA CCG TCA TCT ACA CAG | N/A | This study |
| Acc471_C2 | 471-495 | Competitor for Acc471 | N/A | N/A | AGT CGG GTA CCG TCA TCT ACA CAG | N/A | This study |
| **Acc1011** | **1011-1032** | ***Ca.* Accumulibacter propinquus** | **8/61** | **3** | **GCG AGC ACT CCC AGA TCT CTC** | **40** | **This study** |
| Acc1011_C1 | 1011-1032 | Competitor for Acc1011 | N/A | N/A | GCG AGC ACT CCC AAA TCT CTC | N/A | This study |
| Acc1011_C2 | 1011-1032 | Competitor for Acc1011 | N/A | N/A | TCG AGC ACT CCC AGA TCT CTC | N/A | This study |
| Acc1011_C3 | 1011-1032 | Competitor for Acc1011 | N/A | N/A | GCG GGC ACT CCC AGA TCT CTC | N/A | This study |
| **Acc635** | **635-659** | ***Ca.* Accumulibacter regalis** | **11/61** | **0** | **AAC TCC AGC CTG GCA GTC TCA AAT** | **30** | **This study** |
| Acc635_C1 | 635-659 | Competitor for Acc635 | N/A | N/A | CAC TCC AGC CTG GCA GTC TCA AAT | N/A | This study |
| Acc635_C2 | 635-659 | Competitor for Acc635 | N/A | N/A | AAC TCC AGC CRG GCA GTC TCA AAT | N/A | This study |
| Acc635_C3 | 635-659 | Competitor for Acc635 | N/A | N/A | AAC TCC AGC TTG GCA GTC TCA AAT | N/A | This study |
| **Acc470** | **470-494** | ***Ca.* Accumulibacter aalborgensis and delftensis** | **68/86** | **0** | **TTC GGG TAC CGT CAT CTA CTC AGG** | **30** | **This study** |
| Acc470_C1 | 470-494 | Competitor for Acc470 | N/A | N/A | TGC GGG TAC CGT CAT CTA CTC AGG | N/A | This study |
| Acc470_C2 | 470-494 | Competitor for Acc470 | N/A | N/A | TTC GGG TAC CGT CAT CTA CAC AGG | N/A | This study |
| Acc470_C3 | 470-494 | Competitor for Acc470 | N/A | N/A | TTC GGG TAC CGT CAT CCA CTC AGA | N/A | This study |
| **Acc471_2** | **471-495** | ***Ca.* Accumulibacter iunctus and similis** | **18/21** | **3** | **AGT CGG GTA CCG TCA TCT ACA CAG** | **30** | **This study** |
| Acc471_2_C1 | 471-495 | Competitor for Acc471_2 | N/A | N/A | ATT CGG GTA CCG TCA TCT ACA CAG | N/A | This study |
| Acc471_2_C2 | 471-495 | Competitor for Acc471_2 | N/A | N/A | AGT CGG GTA CCG TCA TCG ACA CAG | N/A | This study |
| **Acc213** | **213- 235** | ***Ca.* Propionivibrio dominans** | **28/32** | **3** | **GGC CGC TCC TAA AGC AAG AGG T** | **35** | **This study** |
| Acc213_C1 | 213- 235 | Competitor for Acc213 | N/A | N/A | GGC CGC TCC CAA AGC AAG AGG T | N/A | This study |
| Acc213_C2 | 213- 235 | Competitor for Acc213 | N/A | N/A | GGC CGC TCC TAA AGC AAC AGG T | N/A | This study |
| **Acc442** | **442-464** | ***Ca.* Proximibacter danicus** | **17/22** | **2** | **GGA GAT GCG ATT TCT TCC CCG C** | **35** | **This study** |
| Acc442_C1 | 442-464 | Competitor for Acc442 | N/A | N/A | GGA GAT GCG ATT TCT TCC CRG | N/A | This study |
| **Acc441** | **441-461** | **midas_s_3472** | **19/34** | **2** | **AGT GCG ATT TCT TCC CCG CC** | **40** | **This study** |
| Acc441_C1 | 441-461 | Competitor for Acc441 | N/A | N/A | AGT GCH ATT TCT TCC CCG CC | N/A | This study |
| Acc441_C2 | 441-461 | Competitor for Acc441 | N/A | N/A | AGT GCG CTT TCT TCC CCG CC | N/A | This study |
| Acc441_C3 | 441-461 | Competitor for Acc441 | N/A | N/A | KGT GCG ATT TCT TCC CCG CC | N/A | This study |
| Acc441_C4 | 441-461 | Competitor for Acc441 | N/A | N/A | AGT GCG ATT TCT TCC CGG CC | N/A | This study |

* Taxonomy and coverage of groups is defined as in the MiDAS4 database. Values given as group hits/ group totals; ** Recommended optimal formamide concentration for use in FISH hybridizations; N/A – not applicable.

**References**

1. Crocetti GR, Hugenholtz P, Bond PL, Schuler A, Keller J, Jenkins D, Blackall LL. 2000. Identification of polyphosphate-accumulating organisms and design of 16S rRNA-directed probes for their detection and quantitation. Appl Environ Microbiol 66:1175–1182.
