# Supplementary material for: Reevaluation of the Phylogenetic Diversity and Global Distribution of the Genus “Candidatus Accumulibacter”
Source: mSystems. 2022 Apr 25;7(3):e00016-22. doi: 10.1128/msystems.00016-22 (PMC9238405; doi:10.1128/msystems.00016-22)
Supplement: TABLE S2 [file msystems.00016-22-s0009.docx]

**Table S2. Comparison of qFISH and 16S rRNA amplicon results (V1-V3).** NA: not applicable.

| **WWTP** | **Sample date** | **Abundance (%)** | |
| --- | --- | --- | --- |
|  |  | **Sequencing** | **qFISH** |
| ***Ca.* Accumulibacter proximus (Acc469)** | | | |
| Odense NE, Denmark | October 2013 | NA | 1.7 ± 0.7 |
| Hjørring, Denmark | August 2011 | NA | <0.5 |
| Aalborg East, Denmark | March 2018 | NA | 0.8 ± 0.5 |
| ***Ca.* Accumulibacter affinis and proximus (Acc471)** | | | |
| Odense NE, Denmark | October 2013 | NA | 1.7 ± 0.6 |
| Hjørring, Denmark | August 2011 | NA | <0.5 |
| Aalborg East, Denmark | March 2018 | NA | 1.1 ± 0.7 |
| ***Ca.* Accumulibacter propinquus (Acc1011)** | | | |
| Odense NE, Denmark | October 2013 | NA | <0.5 |
| Hjørring, Denmark | August 2011 | NA | 0.6 ± 0.3 |
| Aalborg East, Denmark | March 2018 | NA | <0.5 |
| ***Ca.* Accumulibacter phosphatis (Acc635)** | | | |
| Odense NE, Denmark | October 2013 | NA | 1.3 ± 0.5 |
| Hjørring, Denmark | August 2011 | NA | 1.4 ± 0.9 |
| Aalborg East, Denmark | March 2018 | NA | 1 ± 0.5 |
| ***Ca.* Accumulibacter aalborgenesis (Acc470)** | | | |
| Fredericia, Denmark | October 2013 | NA | 4.8 ± 1.6 |
| Esbjerg West, Denmark | August 2013 | NA | 1.1 ± 0.4 |
| Esbjerg East, Denmark | October 2016 | NA | <0.5 |
| ***Ca.* Accumulibacter iunctus and similis (Acc471_2)** | | | |
| Tel Aviv, Israel | March 2018 | 1 | <0.5 |
| Limassol, Cyprus | February 2018 | 1.3 | <0.5 |
| Garmmexwolde, Netherlands | April 2018 | 2.7 | <0.5 |
| ***Ca.* Propionivibrio dominans (Acc213)** | | | |
| Boeslum, Denmark | August 2015 | 2.4 | <0.5 |
| Hjørring, Denmark | November 2018 | 1.4 | <0.5 |
| Piaseczno, Poland | March, 2018 | 2.8 | <0.5 |
| ***Ca.* Proximibacter danicus (Acc442)** | | | |
| Ribe, Denmark | October 2015 | 0.9 | <0.5 |
| East Anglia, England | March 2018 | 0.8 | <0.5 |
| J-town Germany | March 2018 | 0.8 | <0.5 |
| **midas_s_3472 (Acc441)** | | | |
| Esbjerg W, Denmark | February 2017 | 2 | <0.5 |
| Viborg, Denmark | February 2013 | 0.8 | <0.5 |
| Garmmexwolde, Netherlands | April 2018 | 2.7 | <0.5 |
