# Supplementary material for: Reevaluation of the Phylogenetic Diversity and Global Distribution of the Genus “Candidatus Accumulibacter”
Source: mSystems. 2022 Apr 25;7(3):e00016-22. doi: 10.1128/msystems.00016-22 (PMC9238405; doi:10.1128/msystems.00016-22)
Supplement: TEXT S1 [file msystems.00016-22-s0010.docx]

**Additional File 1 – Taxonomic proposal and protologue tables for *Candidatus* species**

Description of “*Candidatus* Accumulibacter regalis” sp. nov.: “*Candidatus* Accumulibacter regalis,” (re.ga´lis, L. masc. adj. *regalis*, royal; indicating the dominating role in the full-scale plants from which the MAG was retrieved). This taxon is represented by the MAG *Candidatus*_Accumulibacter_phosphatis_UW3. The complete protologue can be found in **Table 1.**

Description of “*Candidatus* Accumulibacter appositus” sp. nov.: “*Candidatus* Accumulibacter appositus,” (ap.po´si.tus, L. masc. part. adj. *appositus*, close; indicating the close phylogenetic relationship with *Ca*. Accumulibacter phosphatis). This taxon is represented by the MAG *Candidatus* Accumulibacter sp. BA-92. The complete protologue can be found in **Table 2.**

Description of “*Candidatus* Accumulibacter adiacens” sp. nov.: “*Candidatus* Accumulibacter adiacens,” (ad’ia.cens, L. part. adj. *adiacens*, close; indicating the close phylogenetic relationship with *Ca*. Accumulibacter phosphatis). This taxon is represented by the MAG *Candidatus* Accumulibacter phosphatis HKU1. The complete protologue can be found in **Table 3.**

Description of “*Candidatus* Accumulibacter meliphilus” sp. nov.: “*Candidatus* Accumulibacter meliphilus,” (me.li’phi.lus. L. fem. n. *meles*, badger; Gr. masc. n. *philos*, friend; N.L. masc. n. *meliphilus*, friend of the badger, indicating the badger mascot of the University of Wisconsin-Madison, where the MAG was retrieved). This taxon is represented by the MAG *Candidatus* Accumulibacter UW-LDO. The complete protologue can be found in **Table 4.**

Description of “*Candidatus* Accumulibacter propinquus” sp. nov.: “*Candidatus* Accumulibacter propinquus,” (pro.pin´qu.us. L. masc. adj. propinquus, next of kin; indicating the close phylogenetic relationship with *Ca*. Accumulibacter phosphatis). This taxon is represented by the MAG OdNE_BAT3C.415. The complete protologue can be found in **Table 5**.

Description of “*Candidatus* Accumulibacter contiguus” sp. nov.: “*Candidatus* Accumulibacter contiguus,” (con.ti´gu.us. L. masc. adj. *contiguus*, close; indicating the close phylogenetic relationship with *Ca*. Accumulibacter phosphatis). This taxon is represented by the MAG *Candidatus* Accumulibacter phosphatis SBR_L. The complete protologue can be found in **Table 6**.

Description of “*Candidatus* Accumulibacter vicinus” sp. nov.: “*Candidatus* Accumulibacter vicinus,” (vi.ci´nus. L. masc. adj. *vicinus*, close; indicating the close phylogenetic relationship with *Ca*. Accumulibacter phosphatis). This taxon is represented by the MAG Accumulibacter phosphatis E UBA5574. The complete protologue can be found in **Table 7**.

Description of “*Candidatus* Accumulibacter cognatus” sp. nov.: “*Candidatus* Accumulibacter cognatus,” (co.gna´tus, L. masc. adj. *cognatus*, similar; indicating the close phylogenetic relationship with *Ca*. Accumulibacter phosphatis). This taxon is represented by the MAG *Candidatus* Accumulibacter phosphatis SSA1. The complete protologue can be found in **Table 8**.

Description of “*Candidatus* Accumulibacter affinis” sp. nov.: “*Candidatus* Accumulibacter affinis,” (af.fi´nis. L. masc. adj. *affinis*, next of kin; indicating the close phylogenetic relationship with *Ca*. Accumulibacter phosphatis). This taxon was represented by the MAG Fred_BAT3C.720. The complete protologue can be found in **Table 9**.

Description of “*Candidatus* Accumulibacter proximus” sp. nov.: “*Candidatus* Accumulibacter proximus,” (pro´xi.mus. L. masc. adj. proximus, next of kin; indicating the close phylogenetic relationship with *Ca*. Accumulibacter phosphatis). This taxon was represented by the MAG EsbW_BAT3C.285. The complete protologue can be found in **Table 10**.

Description of “*Candidatus* Accumulibacter necessarius” sp. nov.: “*Candidatus* Accumulibacter necessarius,” (ne.ces.sa´ri.us. L. masc. adj. *necessarius*, next of kin; indicating the close phylogenetic relationship with *Ca*. Accumulibacter phosphatis). This taxon is represented by the MAG *Candidatus* Accumulibacter UW12-POB. The complete protologue can be found in **Table 11**.

Description of “*Candidatus* Accumulibacter iunctus” sp. nov.: “*Candidatus* Accumulibacter iunctus,” (iunc´tus, L. masc. adj. iunctus, next of kin; indicating the close phylogenetic relationship with *Ca*. Accumulibacter phosphatis). This taxon is represented by the MAG Accumulibacter_phosphatis_B UBA2327. The complete protologue can be found in **Table 12**.

Description of “*Candidatus* Accumulibacter adjunctus” sp. nov.: “*Candidatus* Accumulibacter adjunctus,” (ad.iunc´tus. L. masc. part. adj. *adjunctus*, close; indicating the close phylogenetic relationship with *Ca*. Accumulibacter phosphatis). This taxon is represented by the MAG *Candidatus* Accumulibacter sp. SK-12. The complete protologue can be found in **Table 13**.

Description of “*Candidatus* Accumulibacter similis” sp. nov.: “*Candidatus* Accumulibacter similis,” (si´mi.lis. L. masc. adj. *similis*, similar; indicating the close phylogenetic relationship with *Ca*. Accumulibacter phosphatis). This taxon is represented by the MAG *Candidatus* Accumulibacter phosphatis SSB1. The complete protologue can be found in **Table 14**.

Description of “*Candidatus* Accumulibacter conexus” sp. nov.: “*Candidatus* Accumulibacter conexus,” (co.ne´xus. L. masc. part. adj. *conexus*, next of kin; indicating the close phylogenetic relationship with *Ca*. Accumulibacter phosphatis). This taxon is represented by the MAG *Candidatus* Accumulibacter UW7. The complete protologue can be found in **Table 15**.

Description of “*Candidatus* Propionivibrio dominans” sp. nov.: “*Candidatus* Propionivibrio dominans,” (do´mi.nans L. masc. adj. dominans, dominant; indicating the high abundance according to 16S rRNA amplicon sequencing). This taxon is represented by the MAG EsbE_MAXAC.044. The complete protologue can be found in **Table 16**.

Description of “*Candidatus* Proximibacter danicus” gen. nov.: “*Candidatus* Proximibacter danicus,” (pro.xi.mi.bac´ter, from L. masc. adj. proximus, near, and N.L masc. n. bacter, bacterium, N.L masc. n. Proximibacter, indicating a rod-shaped bacterium always found attached to filamentous bacteria; da’ni.cus. L. masc. adj. danicus, Danish). This taxon is represented by the MAG Hirt_BATAC.395. The complete protologue can be found in **Table 17**.

**Table 1. Protologue Table for *Candidatus* Accumulibacter regalis**

| Species name | *Candidatus* Accumulibacter regalis |
| --- | --- |
| Genus name | *Candidatus* Accumulibacter |
| Specific epithet | regalis |
| Type species of the genus | *Candidatus* Accumulibacter phosphatis |
| Genus status | Candidatus |
| Species etymology | Description of “*Candidatus* Accumulibacter regalis” sp. nov.: “*Candidatus* Accumulibacter regalis,” (re.ga´lis, L. masc. adj. regalis, royal; indicating the dominating role in the bioreactors from which the MAG was retrieved). |
| Species status | sp. nov. |
| Designation of the type MAG | GCF_000024165.1 |
| MAG/SAG accession number | IMG accession ID 2687453699 |
| Genome status | High-quality draft |
| Genome size | 4329894 |
| GC mol % | 63.9 |
| Country of origin | USA |
| Region of origin | Wisconsin |
| Source of sample | EBPR bioreactor |
| Geographical location | Madison |
| Latitude | - |
| Longitude | - |
| Depth | - |
| Altitude | - |
| Temperature of the sample | - |
| pH of the sample | - |
| Relationship to oxygen | Facultative anaerobic |
| Energy metabolism | Polyphosphate-accumulating organism |
| Assembly | - |
| Sequencing technology | - |
| Binning software used | - |
| Assembly software used | - |
| Habitat | - |
| Miscellaneous, extraordinary features relevant for the description | - |

**Table 2. Protologue Table for *Candidatus* Accumulibacter appositus**

| Species name | *Candidatus* Accumulibacter appositus |
| --- | --- |
| Genus name | *Candidatus* Accumulibacter |
| Specific epithet | appositus |
| Type species of the genus | *Candidatus* Accumulibacter phosphatis |
| Genus status | Candidatus |
| Species etymology | Description of “*Candidatus* Accumulibacter appositus” sp. nov.: “*Candidatus* Accumulibacter appositus,” (ap.po´si.tus, L. masc. part. adj. *appositus*, close; indicating the close phylogenetic relationship with *Ca*. Accumulibacter phosphatis). This taxon is represented by the MAG *Candidatus* Accumulibacter sp. BA-92. |
| Species status | sp. nov. |
| Designation of the type MAG | GCA_000585055.1 |
| MAG/SAG accession number | GCF_000024165.1 |
| Genome status | High-quality draft |
| Genome size | 4947936 |
| GC mol % | 62.93 |
| Country of origin | Australia |
| Region of origin | - |
| Source of sample | Enriched from a lab-scale EBPR bioreactor |
| Geographical location | - |
| Latitude | 27.4860 |
| Longitude | 153.1907 |
| Depth | - |
| Altitude | - |
| Temperature of the sample | - |
| pH of the sample | - |
| Relationship to oxygen | Facultative anaerobic |
| Energy metabolism | Polyphosphate-accumulating organism |
| Assembly | 1 sample/Scaffold – not sure about this |
| Sequencing technology | Illumina HiSeq |
| Binning software used | - |
| Assembly software used | CLC Genomics Workbench v. 5.5 |
| Habitat | Enriched from a lab-scale EBPR bioreactor |
| Miscellaneous, extraordinary features relevant for the description | - |

**Table 3. Protologue Table for *Candidatus* Accumulibacter adiacens**

| Species name | *Candidatus* Accumulibacter adiacens |
| --- | --- |
| Genus name | *Candidatus* Accumulibacter |
| Specific epithet | adiacens |
| Type species of the genus | *Candidatus* Accumulibacter phosphatis |
| Genus status | Candidatus |
| Species etymology | Description of “*Candidatus* Accumulibacter adiacens” sp. nov.: “*Candidatus* Accumulibacter adiacens,” (ad’ia.cens, L. part. adj. *adiacens*, close; indicating the close phylogenetic relationship with *Ca*. Accumulibacter phosphatis). This taxon is represented by the MAG *Candidatus* Accumulibacter phosphatis HKU1. |
| Species status | sp. nov. |
| Designation of the type MAG | [GCF_000024165.1](https://www.ncbi.nlm.nih.gov/assembly/329571) |
| MAG/SAG accession number | [GCA_000987445.1](https://www.ncbi.nlm.nih.gov/assembly/329571) |
| Genome status | High-quality draft |
| Genome size | 3625256 |
| GC mol % | 63.89 |
| Country of origin | Hong Kong |
| Region of origin | - |
| Source of sample | bioreactor treating saline wastewater (1% salinity) containing high content of P |
| Geographical location | - |
| Latitude | 22.28 |
| Longitude | 114.14 |
| Depth | N/A |
| Altitude | N/A |
| Temperature of the sample | - |
| pH of the sample | N/A |
| Relationship to oxygen | Facultative anaerobe |
| Energy metabolism | Polyphosphate-accumulating organism |
| Assembly | 1 sample |
| Sequencing technology | Illumina HiSeq2000 |
| Binning software used | - |
| Assembly software used | CLC Genomics Workbench v. 6.0.2 |
| Habitat | bioreactor treating saline wastewater (1% salinity) containing high content of P |
| Miscellaneous, extraordinary features relevant for the description | - |

**Table 4. Protologue Table for *Candidatus* Accumulibacter meliphilus**

| Species name | *Candidatus* Accumulibacter meliphilus |
| --- | --- |
| Genus name | *Candidatus* Accumulibacter |
| Specific epithet | meliphilus |
| Type species of the genus | *Candidatus* Accumulibacter phosphatis |
| Genus status | Candidatus |
| Species etymology | Description of “*Candidatus* Accumulibacter meliphilus” sp. nov.: “*Candidatus* Accumulibacter meliphilus,” (me.li’phi.lus. L. fem. n. *meles*, badger; Gr. masc. n. *philos*, friend; N.L. masc. n. *meliphilus*, friend of the badger, indicating the badger mascot of the University of Wisconsin-Madison, where the MAG was retrieved). This taxon is represented by the MAG *Candidatus* Accumulibacter UW-LDO. |
| Species status | sp. nov. |
| Designation of the type MAG | GCF_000024165.1 |
| MAG/SAG accession number | GCA_003332265.1 |
| Genome status | High-quality draft |
| Genome size | 4,701,482 |
| GC mol % | - |
| Country of origin | USA |
| Region of origin | Wisconsin |
| Source of sample | - |
| Geographical location | Madison |
| Latitude | - |
| Longitude | - |
| Depth | - |
| Altitude | - |
| Temperature of the sample | - |
| pH of the sample | - |
| Relationship to oxygen | Facultative anaerobe |
| Energy metabolism | Polyphosphate-accumulating organism |
| Assembly | - |
| Sequencing technology | Illumina HiSeq; Nanopore |
| Binning software used | - |
| Assembly software used | SPAdes v. 3.9.0; Anvi'o v. 4; Links v. 1.5 |
| Habitat | - |
| Miscellaneous, extraordinary features relevant for the description | - |

**Table 5. Protologue Table for *Candidatus* Accumulibacter propinquus**

| Species name | *Candidatus* Accumulibacter propinquus |
| --- | --- |
| Genus name | *Candidatus* Accumulibacter |
| Specific epithet | propinquus |
| Type species of the genus | *Candidatus* Accumulibacter phosphatis |
| Genus status | Candidatus |
| Species etymology | Description of “*Candidatus* Accumulibacter propinquus” sp. nov.: “*Candidatus* Accumulibacter propinquus,” (pro.pin´qu.us L. masc. adj. propinquus, next of kin; indicating the close phylogenetic relationship with *Ca*. Accumulibacter phosphatis). This taxon is represented by the MAG OdNE_BAT3C.415. |
| Species status | sp. nov. |
| Designation of the type MAG | GCF_000024165.1 |
| MAG/SAG accession number | GCA_016714935.1 |
| Genome status | High-quality draft |
| Genome size | 4325360 |
| GC mol % | 63.00 |
| Country of origin | Denmark |
| Region of origin | Odense East |
| Source of sample | Full-scale enriched biological phosphorus removal wastewater treatment plant |
| Geographical location | Odense East |
| Latitude | 55.432604 |
| Longitude | 10.45886 |
| Depth | - |
| Altitude | - |
| Temperature of the sample | Mesophilic |
| pH of the sample | - |
| Relationship to oxygen | Facultative anaerobe |
| Energy metabolism | Polyphosphate-accumulating organism |
| Assembly | 1 sample |
| Sequencing technology | Oxford Nanopore and Illumina Hiseq X |
| Binning software used | MetaBAT2 |
| Assembly software used | CANU v1.8 |
| Habitat | Full-scale enriched biological phosphorus removal wastewater treatment plant |
| Miscellaneous, extraordinary features relevant for the description | Coccoid-shaped cell with a diameter of approx. 0.8-1.2 µm. |

**Table 6. Protologue Table for *Candidatus* Accumulibacter contiguus**

| Species name | *Candidatus* Accumulibacter contiguus |
| --- | --- |
| Genus name | *Candidatus* Accumulibacter |
| Specific epithet | contiguus |
| Type species of the genus | *Candidatus* Accumulibacter phosphatis |
| Genus status | Candidatus |
| Species etymology | Description of “*Candidatus* Accumulibacter contiguus” sp. nov.: “*Candidatus* Accumulibacter contiguus,” (con.ti´gu.us. L. masc. adj. *contiguus*, close; indicating the close phylogenetic relationship with *Ca*. Accumulibacter phosphatis). This taxon is represented by the MAG *Candidatus* Accumulibacter phosphatis SBR_L. |
| Species status | sp. nov. |
| Designation of the type MAG | GCF_000024165.1 |
| MAG/SAG accession number | GCA_012940005.1 |
| Genome status | High-quality draft |
| Genome size | 502444 |
| GC mol % | 61.73 |
| Country of origin | USA |
| Region of origin | Columbia |
| Source of sample | enrichment culture grown in lab-scale EBPR SBR; organism purified/enriched using density-based techniques |
| Geographical location | Columbia |
| Latitude | - |
| Longitude | - |
| Depth | - |
| Altitude | - |
| Temperature of the sample | - |
| pH of the sample | - |
| Relationship to oxygen | Facultative anaerobe |
| Energy metabolism | Polyphosphate-accumulating organism |
| Assembly | - |
| Sequencing technology | IonTorrent |
| Binning software used | - |
| Assembly software used | - |
| Habitat | enrichment culture grown in lab-scale EBPR SBR; organism purified/enriched using density-based techniques |
| Miscellaneous, extraordinary features relevant for the description | - |

**Table 7. Protologue Table for *Candidatus* Accumulibacter vicinus**

| Species name | *Candidatus* Accumulibacter vicinus |
| --- | --- |
| Genus name | *Candidatus* Accumulibacter |
| Specific epithet | vicinus |
| Type species of the genus | *Candidatus* Accumulibacter phosphatis |
| Genus status | Candidatus |
| Species etymology | Description of “*Candidatus* Accumulibacter vicinus” sp. nov.: “*Candidatus* Accumulibacter vicinus,” (vi.ci´nus. L. masc. adj. *vicinus*, close; indicating the close phylogenetic relationship with *Ca*. Accumulibacter phosphatis). This taxon is represented by the MAG Accumulibacter phosphatis UBA5574. |
| Species status | sp. nov. |
| Designation of the type MAG | GCF_000024165.1 |
| MAG/SAG accession number | GCA_002425405.1 |
| Genome status | High-quality draft |
| Genome size | 428324 |
| GC mol % | 61.98 |
| Country of origin | Australia |
| Region of origin | Queensland |
| Source of sample | - |
| Geographical location | Queensland |
| Latitude | - |
| Longitude | - |
| Depth | - |
| Altitude | - |
| Temperature of the sample | - |
| pH of the sample | - |
| Relationship to oxygen | Facultative anaerobe |
| Energy metabolism | Polyphosphate-accumulating organism |
| Assembly | - |
| Sequencing technology | Illumina |
| Binning software used | - |
| Assembly software used | CLC de novo assembler v. 4.4.1 |
| Habitat | - |
| Miscellaneous, extraordinary features relevant for the description | - |

**Table 8. Protologue Table for *Candidatus* Accumulibacter cognatus**

| Species name | *Candidatus* Accumulibacter cognatus |
| --- | --- |
| Genus name | *Candidatus* Accumulibacter |
| Specific epithet | cognatus |
| Type species of the genus | *Candidatus* Accumulibacter phosphatis |
| Genus status | Candidatus |
| Species etymology | Description of “*Candidatus* Accumulibacter cognatus” sp. nov.: “*Candidatus* Accumulibacter cognatus,” (co.gna´tus, L. masc. adj. *cognatus*, similar; indicating the close phylogenetic relationship with *Ca*. Accumulibacter phosphatis). This taxon is represented by the MAG *Candidatus* Accumulibacter phosphatis SSA1. |
| Species status | sp. nov. |
| Designation of the type MAG | GCF_000024165.1 |
| MAG/SAG accession number | GCA_013414765.1 |
| Genome status | High-quality draft |
| Genome size | 516951 |
| GC mol % | 61.4 |
| Country of origin | Singapore |
| Region of origin | Singapore |
| Source of sample | Activated sludge, continuous culture bioreactor |
| Geographical location | Jurong West |
| Latitude | 1.344722 |
| Longitude | 103.681389 |
| Depth | - |
| Altitude | - |
| Temperature of the sample | - |
| pH of the sample | - |
| Relationship to oxygen | Facultative anaerobe |
| Energy metabolism | Polyphosphate-accumulating organism |
| Assembly | 1 sample |
| Sequencing technology | Oxford Nanopore MiniION |
| Binning software used | - |
| Assembly software used | Canu v. 1.8 |
| Habitat | Activated sludge, continuous culture bioreactor |
| Miscellaneous, extraordinary features relevant for the description | - |

**Table 9. Protologue Table for *Candidatus* Accumulibacter affinis**

| Species name | *Candidatus* Accumulibacter affinis |
| --- | --- |
| Genus name | *Candidatus* Accumulibacter |
| Specific epithet | affinis |
| Type species of the genus | *Candidatus* Accumulibacter phosphatis |
| Genus status | Candidatus |
| Species etymology | Description of “*Candidatus* Accumulibacter affinis” sp. nov.: “*Candidatus* Accumulibacter affinis,” (af.fi´nis. L. masc. adj. affinis, next of kin; indicating the close phylogenetic relationship with *Ca*. Accumulibacter phosphatis). This taxon is represented by the MAG Fred_BAT3C.720. |
| Species status | sp. nov. |
| Designation of the type MAG | GCF_000024165.1 |
| MAG/SAG accession number | GCA_016713625.1 |
| Genome status | High-quality draft |
| Genome size | 5041483 |
| GC mol % | 62.30 |
| Country of origin | Denmark |
| Region of origin | Fredericia |
| Source of sample | Full-scale enriched biological phosphorus removal wastewater treatment plant |
| Geographical location | Fredericia |
| Latitude | 55.552368 |
| Longitude | 9.720404 |
| Depth | - |
| Altitude | - |
| Temperature of the sample | Mesophilic |
| pH of the sample | - |
| Relationship to oxygen | Facultative anaerobe |
| Energy metabolism | Polyphosphate-accumulating organism |
| Assembly | 1 sample |
| Sequencing technology | Oxford Nanopore and Illumina Hiseq X |
| Binning software used | MetaBAT2 |
| Assembly software used | CANU v1.8 |
| Habitat | Full-scale enriched biological phosphorus removal wastewater treatment plant |
| Miscellaneous, extraordinary features relevant for the description | Coccoid-shaped cells with a diameter approx. of 0.5-0.7 µm |

**Table 10. Protologue Table for *Candidatus* Accumulibacter proximus**

| Species name | *Candidatus* Accumulibacter proximus |
| --- | --- |
| Genus name | *Candidatus* Accumulibacter |
| Specific epithet | proximus |
| Type species of the genus | *Candidatus* Accumulibacter phosphatis |
| Genus status | Candidatus |
| Species etymology | Description of “*Candidatus* Accumulibacter proximus” sp. nov.: “*Candidatus* Accumulibacter proximus,” (pro´xi.mus L. masc. adj. proximus, next of kin; indicating the close phylogenetic with *Ca*. Accumulibacter phosphatis). This taxon is represented by the MAG EsbW_BATAC.285. |
| Species status | sp. nov. |
| Designation of the type MAG | GCF_000024165.1 |
| MAG/SAG accession number | GCA_016709675.1 |
| Genome status | High-quality draft |
| Genome size | 5402764 |
| GC mol % | 62.70 |
| Country of origin | Denmark |
| Region of origin | Esbjerg West |
| Source of sample | Full-scale biological nutrient removal wastewater treatment plant |
| Geographical location | Esbjerg West |
| Latitude | 55.488097 |
| Longitude | 8.430505 |
| Depth | - |
| Altitude | - |
| Temperature of the sample | Mesophilic |
| pH of the sample | - |
| Relationship to oxygen | Facultative anaerobe |
| Energy metabolism | Polyphosphate-accumulating organism |
| Assembly | 1 sample |
| Sequencing technology | Oxford Nanopore and Illumina Hiseq X |
| Binning software used | MetaBAT2 |
| Assembly software used | CANU v1.8 |
| Habitat | Full-scale biological nutrient removal wastewater treatment plant |
| Miscellaneous, extraordinary features relevant for the description | Coccoid-shaped cells with a diameter approx. of 0.5-0.7 µm |

**Table 11. Protologue Table for *Candidatus* Accumulibacter necessarius**

| Species name | *Candidatus* Accumulibacter necessarius |
| --- | --- |
| Genus name | *Candidatus* Accumulibacter |
| Specific epithet | necessarius |
| Type species of the genus | *Candidatus* Accumulibacter phosphatis |
| Genus status | Candidatus |
| Species etymology | Description of “*Candidatus* Accumulibacter necessarius” sp. nov.: “*Candidatus* Accumulibacter necessarius,” (ne.ces.sa´ri.us. L. masc. adj. *necessarius*, next of kin; indicating the close phylogenetic relationship with *Ca*. Accumulibacter phosphatis). This taxon is represented by the MAG *Candidatus* Accumulibacter UW12-POB. |
| Species status | sp. nov. |
| Designation of the type MAG | GCF_000024165.1 |
| MAG/SAG accession number | GCA_017302435.1 |
| Genome status | High-quality draft |
| Genome size | 456421 |
| GC mol % | 62.7 |
| Country of origin | USA |
| Region of origin | Wisconsin |
| Source of sample | Activated sludge |
| Geographical location | Madison |
| Latitude | - |
| Longitude | - |
| Depth | - |
| Altitude | - |
| Temperature of the sample | - |
| pH of the sample | - |
| Relationship to oxygen | Facultative anaerobe |
| Energy metabolism | Polyphosphate-accumulating organism |
| Assembly | 1 sample |
| Sequencing technology | Illumina HiSeq |
| Binning software used | - |
| Assembly software used | metaSPAdes v. 3.9.0 |
| Habitat | Activated sludge |
| Miscellaneous, extraordinary features relevant for the description | - |

**Table 12. Protologue Table for *Candidatus* Accumulibacter iunctus**

| Species name | *Candidatus* Accumulibacter iunctus |
| --- | --- |
| Genus name | *Candidatus* Accumulibacter |
| Specific epithet | iunctus |
| Type species of the genus | *Candidatus* Accumulibacter phosphatis |
| Genus status | Candidatus |
| Species etymology | Description of “*Candidatus* Accumulibacter iunctus” sp. nov.: “*Candidatus* Accumulibacter iunctus,” (iunc´tus L. masc. adj. iunctus, next of kin; indicating the close phylogenetic relationship with *Ca*. Accumulibacter phosphatis). This taxon is represented by the MAG *Candidatus* Accumulibacter UBA2327. |
| Species status | sp. nov. |
| Designation of the type MAG | GCF_000024165.1 |
| MAG/SAG accession number | GCA 002345025.1 |
| Genome status | High-quality draft |
| Genome size | 4431027 |
| GC mol % | 65.20 |
| Country of origin | Australia |
| Region of origin | Queensland |
| Source of sample | Bioreactor sludge metagenome |
| Geographical location | Brisbane, Thorneside Wastewater Treatment Plant |
| Latitude | - |
| Longitude | - |
| Depth | - |
| Altitude | - |
| Temperature of the sample | - |
| pH of the sample | - |
| Relationship to oxygen | Facultative anaerobe |
| Energy metabolism | Polyphosphate-accumulating organism |
| Assembly | 1 sample |
| Sequencing technology | Illumina HiSeq |
| Binning software used | - |
| Assembly software used | CLC de novo assembler v. 4.4.1 |
| Habitat | Activated sludge |
| Miscellaneous, extraordinary features relevant for the description | - |

**Table 13. Protologue Table for *Candidatus* Accumulibacter adjunctus**

| Species name | *Candidatus* Accumulibacter adjunctus |
| --- | --- |
| Genus name | *Candidatus* Accumulibacter |
| Specific epithet | adiunctus |
| Type species of the genus | *Candidatus* Accumulibacter phosphatis |
| Genus status | Candidatus |
| Species etymology | Description of “Candidatus Accumulibacter adjunctus” sp. nov.: “Candidatus Accumulibacter adjunctus,” (ad.iunc´tus. L. masc. part. adj. adjunctus, close; indicating the close phylogenetic relationship with Ca. Accumulibacter phosphatis). This taxon is represented by the MAG *Candidatus* Accumulibacter sp. SK-12 |
| Species status | sp. nov. |
| Designation of the type MAG | GCF_000024165.1 |
| MAG/SAG accession number | GCA_000585015.1 |
| Genome status | High-quality draft |
| Genome size | 4,412,715 |
| GC mol % | 65.80 |
| Country of origin | Australia |
| Region of origin | Queensland |
| Source of sample | synthetic wastewater |
| Geographical location | Brisbane, Thorneside Wastewater Treatment Plant |
| Latitude | 27.485973 |
| Longitude | 153.190699 |
| Depth | - |
| Altitude | - |
| Temperature of the sample | - |
| pH of the sample | - |
| Relationship to oxygen | Facultative anaerobe |
| Energy metabolism | Polyphosphate-accumulating organism |
| Assembly | - |
| Sequencing technology | Illumina HiSeq |
| Binning software used | - |
| Assembly software used | CLC Genomics Workbench v. 5.5 |
| Habitat | EBPR sludge |
| Miscellaneous, extraordinary features relevant for the description | - |

**Table 14. Protologue Table for *Candidatus* Accumulibacter similis**

| Species name | *Candidatus* Accumulibacter similis |
| --- | --- |
| Genus name | *Candidatus* Accumulibacter |
| Specific epithet | similis |
| Type species of the genus | *Candidatus* Accumulibacter phosphatis |
| Genus status | Candidatus |
| Species etymology | Description of “*Candidatus* Accumulibacter similis” sp. nov.: “*Candidatus* Accumulibacter similis,” (si´mi.lis. L. masc. adj. *similis*, similar; indicating the close phylogenetic relationship with *Ca*. Accumulibacter phosphatis). This taxon is represented by the MAG *Candidatus* Accumulibacter phosphatis SSB1. |
| Species status | sp. nov. |
| Designation of the type MAG | GCF_000024165.1 |
| MAG/SAG accession number | GCA_013347225.1 |
| Genome status | High-quality draft |
| Genome size | 503901 |
| GC mol % | 66.0 |
| Country of origin | Singapore |
| Region of origin | Jurong West |
| Source of sample | Activated sludge |
| Geographical location | Jurong West |
| Latitude | 1.344722 |
| Longitude | 103.681389 |
| Depth | - |
| Altitude | - |
| Temperature of the sample | - |
| pH of the sample | - |
| Relationship to oxygen | Facultative anaerobe |
| Energy metabolism | Polyphosphate-accumulating organism |
| Assembly | 1 sample |
| Sequencing technology | Oxford Nanopore MiniION |
| Binning software used | - |
| Assembly software used | Canu v. 1.8 |
| Habitat | Activated sludge |
| Miscellaneous, extraordinary features relevant for the description | - |

**Table 15. Protologue Table for *Candidatus* Accumulibacter conexus**

| Species name | *Candidatus* Accumulibacter conexus |
| --- | --- |
| Genus name | *Candidatus* Accumulibacter |
| Specific epithet | conexus |
| Type species of the genus | *Candidatus* Accumulibacter phosphatis |
| Genus status | Candidatus |
| Species etymology | Description of “*Candidatus* Accumulibacter conexus” sp. nov.: “*Candidatus* Accumulibacter conexus,” (co.ne´xus. L. masc. part. adj. *conexus*, next of kin; indicating the close phylogenetic relationship with *Ca*. Accumulibacter phosphatis). This taxon is represented by the MAG *Candidatus* Accumulibacter UW7 |
| Species status | sp. nov. |
| Designation of the type MAG | GCF_000024165.1 |
| MAG/SAG accession number | GCA_017592775.1 |
| Genome status | High-quality draft |
| Genome size | 487076 |
| GC mol % | 66.30 |
| Country of origin | USA |
| Region of origin | Wisconsin |
| Source of sample | Activate sludge |
| Geographical location | Wisconsin |
| Latitude | 43.071 |
| Longitude | 89.401 |
| Depth | - |
| Altitude | - |
| Temperature of the sample | - |
| pH of the sample | - |
| Relationship to oxygen | Facultative anaerobe |
| Energy metabolism | Polyphosphate-accumulating organism |
| Assembly | 1 sample |
| Sequencing technology | Illumina HiSeq |
| Binning software used | - |
| Assembly software used | SPAdes v. 3.9.0 |
| Habitat | Activated sludge |
| Miscellaneous, extraordinary features relevant for the description | - |

**Table 16. Protologue Table for *Candidatus* Propionivibrio dominans**

| Species name | *Candidatus* Propionivibrio dominans |
| --- | --- |
| Genus name | *Candidatus* Propionivibrio |
| Specific epithet | dominans |
| Type species of the genus | *Propionivibrio dicarboxylicus* |
| Genus status | Candidatus |
| Species etymology | Description of “*Candidatus* Propionivibrio dominans” sp. nov.: “*Candidatus* Propionivibrio dominans,” (do´mi.nans L. masc. adj. dominans, dominant; indicating the high abundance according to 16S rRNA amplioon sequencing). This taxon is represented by the MAG EsbW_MAXAC.044 |
| Species status | sp. nov. |
| Designation of the type MAG | GCA_900099695.1 |
| MAG/SAG accession number | GCA_016709335.1 |
| Genome status | High-quality draft |
| Genome size | 4421984 |
| GC mol % | 58.50 |
| Country of origin | Denmark |
| Region of origin | Esbjerg West |
| Source of sample | Full-scale biological nutrient removal wastewater treatment plant |
| Geographical location | Esbjerg West |
| Latitude | 55.488097 |
| Longitude | 8.430505 |
| Depth | - |
| Altitude | - |
| Temperature of the sample | Mesophilic |
| pH of the sample | - |
| Relationship to oxygen | Facultative anaerobe |
| Energy metabolism | Glycogen-accumulating organism |
| Assembly | 1 sample |
| Sequencing technology | Oxford Nanopore and Illumina Hiseq X |
| Binning software used | MetaBAT2 |
| Assembly software used | CANU v1.8 |
| Habitat | Full-scale biological nutrient removal wastewater treatment plant |
| Miscellaneous, extraordinary features relevant for the description | Rod-shaped cell, with size 0.5-0.6 × 0.9-1.1 µm |

**Table 17. Protologue Table for *Candidatus* Proximibacter danicus**

| Species name | *Candidatus* Proximibacter danicus |
| --- | --- |
| Genus name | *Candidatus* Proximibacter |
| Specific epithet | danicus |
| Type species of the genus | *Candidatus* Proximibacter daniensis |
| Genus status | Candidatus |
| Species etymology | Description of “*Candidatus* Proximibacter danicus” gen. nov.: “*Candidatus* Proximibacter danicus,” (pro.xi.mi.bac´ter, from L. masc. adj. proximus, near, and N.L masc. n. bacter, bacterium, N.L masc. n. Proximibacter, indicating a rod-shaped bacterium always found attached to filamentous bacteria; da’ni.cus. L. masc. adj. danicus, Danish). This taxon is represented by the MAG Hirt_BATAC.395. |
| Species status | sp. nov. |
| Designation of the type MAG | GCA_016710885.1 |
| MAG/SAG accession number | GCA_016710885.1 |
| Genome status | High-quality draft |
| Genome size | 3246725 |
| GC mol % | 58.60 |
| Country of origin | Denmark |
| Region of origin | Hirtshals |
| Source of sample | Full-scale enriched biological phosphorus removal wastewater treatment plant |
| Geographical location | Hirtshals |
| Latitude | 57.577275 |
| Longitude | 9.992971 |
| Depth | N/A |
| Altitude | N/A |
| Temperature of the sample | Mesophilic |
| pH of the sample | N/A |
| Relationship to oxygen | - |
| Energy metabolism | - |
| Assembly | 1 sample |
| Sequencing technology | Oxford Nanopore and Illumina Hiseq X |
| Binning software used | MetaBAT2 |
| Assembly software used | CANU v1.8 |
| Habitat | Full-scale enriched biological phosphorus removal wastewater treatment plant |
| Miscellaneous, extraordinary features relevant for the description | Rod-shaped cells with size 0.3-05 × 1-2 µm, often attached to filamentous bacteria |
